# Exploration of fecal microbiota in newly diagnosed patients with inflammatory bowel disease using shotgun metagenomics

**DOI:** 10.3389/fcimb.2025.1595884

**Published:** 2025-07-01

**Authors:** Macarena Orejudo, Manuel J. Gómez, Sabino Riestra, Montserrat Rivero, Ana Gutiérrez, Iago Rodríguez-Lago, Luis Fernández-Salazar, Daniel Ceballos, José Manuel Benítez, Mariam Aguas, Iria Bastón-Rey, Fernando Bermejo, María José Casanova, Rufo H. Lorente-Poyatos, Yolanda Ber, Daniel Ginard, María Esteve, Ruth de Francisco, María José García, Rubén Francés, Ainhoa Rodríguez, Noelia Alcaide Suárez, Elena Guerra del Río, Pilar Soto, Pilar Nos, Manuel Barreiro-de Acosta, Iván Guerra, Daniel Hervías Cruz, Manuel Domínguez Cajal, Vanesa Royo, Montserrat Aceituno, Laila Aldars-García, Ana Garre, Cristina Ramírez, Irene Soleto, Ina Schuppe-Koistinen, Lars Engstrand, Montse Baldán-Martín, Fátima Sánchez-Cabo, Javier P. Gisbert, María Chaparro

**Affiliations:** ^1^ Hospital Universitario de La Princesa, Instituto de Investigación Sanitaria Princesa (IIS-Princesa), Universidad Autónoma de Madrid (UAM), and Centro de Investigación Biomédica en Red de Enfermedades Hepáticas y Digestivas (CIBEREHD), Madrid, Spain; ^2^ Centro Nacional de Investigaciones Cardiovasculares Carlos III (CNIC), Madrid, Spain; ^3^ Gastroenterology Department, Hospital Universitario Central de Asturias and Instituto de Investigación Sanitaria del Principado de Asturias (ISPA), Asturias, Spain; ^4^ Gastroenterology Department, Hospital Universitario Marqués de Valdecilla, Grupo de Investigación Clínica y Traslacional en Enfermedades Digestivas, Instituto de Investigación Valdecilla (IDIVAL), Santander, Spain; ^5^ Hospital General Universitario Dr Balmis de Alicante, Instituto de Investigación Sanitaria y Biomédica de Alicante (ISABIAL) y Centro de Investigación Biomédica en Red de Enfermedades Hepáticas y Digestivas (CIBEREHD), Alicante, Spain; ^6^ Gastroenterology Department, Hospital Universitario de Galdakao, Galdakao, Vizcaya, Spain; ^7^ Biobizkaia Health Research Institute, Galdakao, Vizcaya, Spain; ^8^ Gastroenterology Department, Hospital Clínico Universitario de Valladolid, Universidad de Valladolid, Valladolid, Spain; ^9^ Gastroenterology Department, Hospital Universitario de Gran Canaria Dr. Negrín, Las Palmas de Gran Canaria, Spain; ^10^ Gastroenterology Department, Hospital Universitario Reina Sofía and Instituto Maimónides de Investigación Biomédica de Córdoba (IMIBIC), Córdoba, Spain; ^11^ Hospital Universitario La Fe, Health Research Institute La Fe, Valencia, Spain; ^12^ Complexo Hospitalario Universitario de Santiago, Santiago de Compostela, Spain; ^13^ Hospital Universitario de Fuenlabrada, Madrid, Spain; ^14^ Hospital General Universitario de Ciudad Real, Ciudad Real, Spain; ^15^ Hospital San Jorge, Huesca, Spain; ^16^ Hospital Universitari Son Espases, Palma de Mallorca, Spain; ^17^ Hospital Universitari Mutua Terrassa, Terrassa, Spain. Centro de Investigación Biomédica en Red de Enfermedades Hepáticas y Digestivas (CIBEREHD), Madrid, Spain; ^18^ Grupo de Inmunobiología Hepática e Intestinal, Dpto. Medicina Clínica e Instituto Instituto de Investigación, Desarrollo e Innovación en Biotecnología Sanitaria de Elche (IDIBE), Universidad Miguel Hernández, San Juan, Alicante, Spain; ^19^ Centre for Translational Microbiome Research, Department Microbiology, Tumor and Cell Biology, Karolinska Institute, Solna, Sweden

**Keywords:** inflammatory bowel disease, Crohn’s disease, ulcerative colitis, microbiota, metagenomics, shotgun

## Abstract

**Introduction:**

Dysbiosis is a key mechanism in inflammatory bowel disease (IBD) pathophysiology. Previous microbiota studies in IBD generally have involved patients treated with immunosuppressive agents, which can affect the results. We aimed to elucidate the fecal microbiota composition in newly diagnosed treatment-naïve IBD patients.

**Methods:**

Microbiota from stool samples were investigated using shotgun metagenomics sequencing and subsequent bioinformatics analysis.

**Results:**

A total of 103 patients with Crohn's disease (CD), 144 with ulcerative colitis (UC), and 49 healthy controls (HC) were included. CD patients had significantly lower species-level diversity than those with UC and HC. CD subgroups with Ileocolonic location and stricturing behavior showed reduced diversity compared to HC. A negative correlation was observed between endoscopic severity and microbial diversity in CD patients. UC patients had similar microbial diversity to HC, which was unaffected by disease activity. Taxonomic abundance analysis revealed a tendency towards a higher relative abundance of Escherichia coli and a lower relative abundance of Faecalibacterium prausnitzii in IBD patients compared to HC. However, the most significant differences in these patients compared to HC were observed in less abundant species, such as Toxoplasma gondii, Gemella morbillorum, and several species of the Adlercreutzia genera. Functional analysis in these patients highlighted changes in carbohydrate and nucleotide pathways.

**Discussion:**

Our data suggest that newly diagnosed CD patients show significant microbiota composition disparities compared to UC patients and HC. Microbiota differences in these patients are linked to dysbiosis, characterized by a reduction in beneficial genera such as Gemella and Adlercreutzia, and a rise in pathogenic species.

## 1 Introduction

Inflammatory bowel disease (IBD) comprises chronic gastrointestinal disorders typified by inflammation within the gastrointestinal tract, notably Crohn’s disease (CD) and ulcerative colitis (UC) (Tavakoli et al., 2021; Elhag et al., 2022). Currently, there is no curative treatment for IBD, and the treatment goal is mainly to control the chronic inflammation in order to avoid the complications associated with this disease (Majchrzak and Fichna, 2019). Additionally, although it can be detected at any age, it is typically diagnosed in young adults, significantly impacting their quality of life. IBD is a global issue across different healthcare systems, since its prevalence is constantly rising in many developed countries. The gold standard for IBD diagnosis remains endoscopic evaluation with histological confirmation, which allows direct assessment of mucosal inflammation and disease extent (Kucharzik et al., 2021). Consequently, there is an increasing urgency to devise novel approaches enabling the identification of non-invasive biomarkers with potential diagnostic, prognostic, and disease-monitoring applications suitable for clinical practice.

While CD and UC share some therapies, their treatment strategies differ notably. Both follow a step-up approach using 5-aminosalicylates (5-ASAs), corticosteroids, immunomodulators, and biologics (Vieujean et al., 2025). However, 5-ASAs are mainly effective in UC, with limited benefit in CD (Raine et al., 2022). Exclusive enteral nutrition is a key first-line therapy in pediatric CD (Narula et al., 2018). About 70-80% of CD patients need surgery over their lifetime, often for complications, while 20-30% of UC patients require colectomy, which is curative only in UC (Adamina et al., 2020; Spinelli et al., 2022). CD treatment also considers disease location and behavior, whereas UC management depends on extent and severity (Lamb et al., 2019).

Microbiota include the diverse community of living microorganisms found in a particular environment, such as the gastrointestinal tract (Hou et al., 2022). The intestinal microbiota and the host are in symbiotic relationship, in which the microbiota play a crucial role in processes such as the maintenance of homeostasis, carbohydrate and vitamin metabolism, and the development of the immune system (Aldars‐garcía et al., 2021; Shan et al., 2022).

Evidence indicates that bacteria play a pivotal role in the pathophysiology of IBD through an imbalance in the gut microbiota (Park et al., 2020). Yet, it remains unclear whether dysbiosis is the cause or consequence of an altered immune response within the intestinal mucosa (Guo et al., 2022). The intestinal milieu, including the microbiota and their byproducts, significantly impacts bowel homeostasis (Fernández-Tomé et al., 2021). Broadly, IBD correlates with diminished microbial diversity in the gut, marked by decreased *Firmicutes* phylum and heightened proportions of *Proteobacteria* (Mah et al., 2023). Beyond bacteria, scientific evidence suggests that viruses, archaea, and fungi might also contribute significantly to establishing diverse microbial communities in the intestine and to the etiology of IBD (Sartor and Wu, 2017; Ungaro et al., 2019).

Progress in DNA sequencing techniques has unveiled the crucial pathogenic role of the microbial community in IBD development. However, studies often involve patients undergoing medical therapy, mainly including immunosuppressive drugs, which can alter the microbiota and complicate result interpretation (Doherty et al., 2018; Ribaldone et al., 2019; Kowalska-Duplaga et al., 2020). Furthermore, in studies examining the intestinal microbiota, cohorts often tend to be small (Ricanek et al., 2012; Nishino et al., 2018; Dovrolis et al., 2020). Also frequently, only the bacterial composition is analyzed, leaving the rest of the microbial domains unstudied (Ricanek et al., 2012; Nishino et al., 2018; Altomare et al., 2019; Ribaldone et al., 2019; Dovrolis et al., 2020; Kowalska-Duplaga et al., 2020).

In recent years, shotgun metagenomics has emerged as a revolutionary tool for studying microbial diversity and function in different environments, including the human gut. This technique allows for an unbiased and comprehensive analysis of the gut microbiota by sequencing DNA extracted from fecal samples, providing a more detailed insight into this microbial community and its potential implications for health and disease (Franzosa et al., 2015).

The aim of this study was to analyze the composition of fecal microbiota in adult patients recently diagnosed with IBD, before starting any treatment, using shotgun metagenome sequencing. The use of an exclusive cohort, both in terms of its high number of patients and its thorough clinical information, makes it possible to describe fecal microbiota composition at IBD diagnosis, when the disease has not been modified by drugs. Hopefully, our study will constitute a starting point for analyzing the relationship between the fecal microbiota and IBD.

## 2 Materials and methods

### 2.1 Study population

The research was conducted at the Gastroenterology Department of Hospital Universitario de La Princesa (Madrid, Spain). The study included patients diagnosed with IBD, specifically those with CD and UC, who were part of the IBDomics cohort. IBDomics is a prospective, observational, multicenter, population-based study focused on newly diagnosed IBD cases in Spain, with data collected over 18 months. The recruitment process began on October 2017 and ended on April 2019, involving 16 Spanish hospitals. The current study specifically involved the metagenomics analysis of stool samples collected from these patients.

Prior to sample collection, all participants signed an informed consent form, and all procedures adhered to the approved study protocol. Personal anonymized data of participants were stored electronically, with researchers strictly adhering to prescribed procedures for safeguarding personal data.

### 2.2 Patients and samples

Adult patients newly diagnosed with either CD or UC (less than one month from the diagnosis) were prospectively included. Patients who had started any treatment before the baseline visit, those receiving immunosuppressive therapy for conditions other than IBD, those with immune-mediated diseases different from IBD, those who had cancer or an active infection, and pregnant or breastfeeding women were excluded.

Demographic data were obtained from each participant. The variables recorded in the study database included: sex, smoking habits, date and age at IBD diagnosis, type of IBD (location and behavior), clinical and endoscopic activity, and presence of extraintestinal manifestations.

Part of the study was conducted during the COVID-19 pandemic, when access to human samples was limited. For this reason, samples and data from healthy controls (HC) included in this study were managed and provided by La Fe Biobank (PT17/0015/0043) with the approval of both the Ethics and the Scientific Committees and have been processed following standard procedures.

Stool samples were obtained either at the baseline visit day or within 24 hours before the visit (within one month after the diagnosis, before starting any treatment for IBD). They were stored in a refrigerator (maintained between 3°C and 5°C) until they were sent to the central laboratory. Samples were collected and conserved using DNA/RNA Shield Fecal Collection tubes (Zymo Research, Irvine, CA), and they were finally frozen at −80°C until use.

### 2.3 Shotgun metagenome analysis

A total of 363 fecal samples were initially collected. DNA extraction, library preparation and sequencing were performed at the Centre for Translational Microbiome Research (CTMR), Karolinska Institutet (Stockholm, Sweden). DNA sequencing libraries were prepared using the MGIEasy FS DNA Library Prep Set. Equimolarly pooled libraries were subjected to whole metagenome shotgun sequencing using the DNBSEQ-G400 platform (MGI Tech Co., Ltd., Shenzhen, China). Microbial community and DNA standards (Zymo Research, Irvine, CA) were included to assess bias and errors before and after the step of nucleic acid purification. Sixteen samples failed sequencing and were excluded from further analysis.

The average sequencing depth was approximately 58 million, 100 nucleotide long, paired-end reads per sample.

### 2.4 Bioinformatic and statistical analyses

347 samples with successful sequencing were initially used for bioinformatic analyses. Sequencing reads were pre-processed by means of a pipeline that used FastQC (Andrews, 2010), to assess read quality, and Cutadapt (Martin, 2011) to eliminate Illumina adapter contaminations, to remove the first 6 nt, to trim 3’-ends by quality (q=20), and to discard reads that were shorter than 50 base pairs. Next, reads were classified with Kraken2 (Wood et al., 2019) against a Homo sapiens reference, to eliminate human reads. A total of 34 samples, most of them corresponding to UC cases, were found to contain more than 75% human reads. Resulting reads were then classified with Kraken2/Bracken (Lu et al., 2017) against a database containing sequences from archaea, bacteria, virus, plasmids, human, UniVec_Core, protozoa and fungi, constructed with kraken-build on 2023-03-13. The detailed information of this database is provided in **Supplementary Table 1**. Finally, 51 samples with a total length of classified sequenced fragments lower than 2 Gb were eliminated. Therefore, 296 samples (247 from IBD patients and 49 from healthy controls) with adequate sequencing depth were retained for downstream metagenomic analyses.

Sample reports, describing Bracken-corrected counts at each taxonomic level, were then processed with an R script to filter and normalize the data and to produce exploratory plots. For each sample, any feature with raw abundance lower than 10 was excluded. Diversity indices were calculated with the vegan package (Oksanen et al., 2022), using Shannon index (α-diversity) (Shannon, 1948) and Bray-Curtis dissimilarity (β-diversity) (Bray and Curtis, 1957) to describe within-group diversity and to quantify microbiome distance between groups, respectively.

The tool HUMAnN3 was applied for functional profiling (Franzosa et al., 2018), which infers metabolic pathway abundances based on the presence and relative abundance of genes encoding enzymes within the metagenomic data. Therefore, these data represent the genomic potential of the microbial community to carry out specific metabolic functions.

Differential taxon and metabolic pathway abundances across multiple groups was tested with Kruskal-Wallis. Pairwise tests were performed with limma (Ritchie et al., 2015), focusing on groups with at least one count per million in at least 10 samples. Statistical analyses were performed in RStudio version 4.3.1. Correlation plots were generated in RStudio with the addition of correlation coefficient and p-value to indicate significance.

## 3 Results

### 3.1 Clinical characteristics

Microbiota analysis was carried out on fecal samples obtained from 247 patients diagnosed with IBD (103 with CD and 144 with UC) and 49 HC. Main characteristics of the included patients are summarized in **Table 1**. Half of the patients with CD exhibited ileal location, and 91% had inflammatory behavior. No significant differences in age or sex were observed between IBD patients and HC.

**Table 1:** Demographic and clinical characteristics of the study groups.

Characteristics	HC (n=49)	CD (n=103)	UC (n=144)
Female gender, n (%)	27 (55)	50 (49)	72 (50)
Non-smokers at diagnosis, n (%)	25 (51)	85 (83)	128 (89)
Median age, years (range)	40 (24-42)	43 (22-88)	48 (23-87)
CD characteristics			
Location			
L1: ileal (%)		59 (57)	
L2: colonic (%)		16 (16)	
L3: ileocolonic (%)		28 (27)	
Behavior			
B1: inflammatory (%)		91 (88)	
B2: stricturing (%)		7 (7)	
B3: penetrating (%)		5 (5)	
Endoscopic activity (SES-CD)			
Mild, 3-6 (%)		61 (60)	
Moderate, 7-15 (%)		28 (27)	
Severe, ≥ 16 (%)		14 (13)	
UC characteristics			
Extension			
E1: Proctitis (%)			57 (40)
E2: Left sided (%)			59 (41)
E3: Extensive (%)			28 (19)
Endoscopic activity (Endoscopic Mayo index)			
Mild (%)			34 (24)
Moderate (%)			91 (63)
Severe (%)			19 (13)

HC, healthy controls; CD, Crohn´s disease; UC, ulcerative colitis; SES-CD, Simple endoscopic score for Crohn’s disease. Values in parentheses indicate the percentage of each characteristic relative to the total number of patients in each group (HC, CD, and UC), except for median age, where the numbers in parentheses indicate the age range.

**Figure 1:**
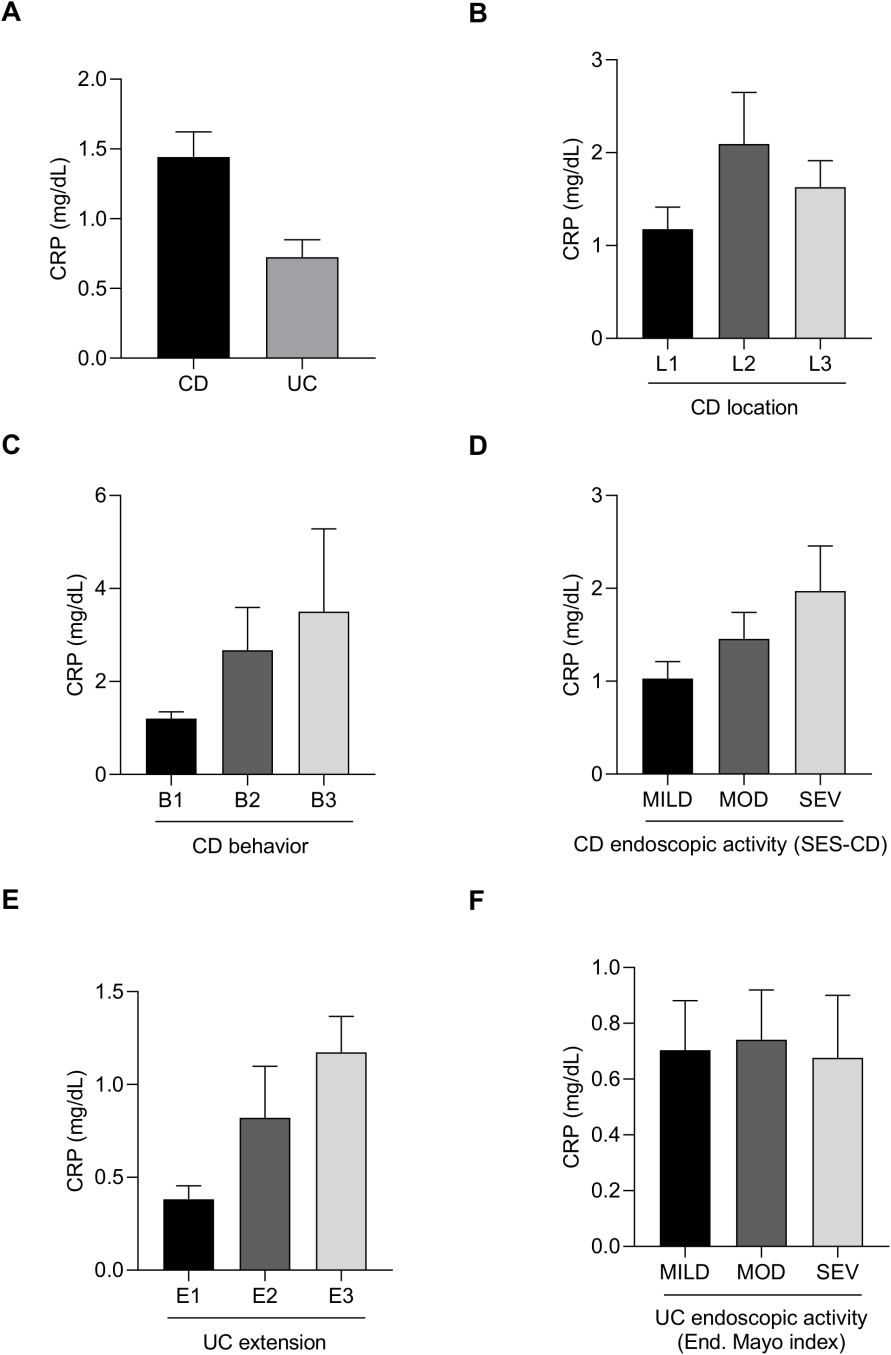
Serum C-reactive protein (CRP) levels in patients with newly diagnosed inflammatory bowel disease. Bar charts showing C-reactive protein (CRP) levels in CD and UC patients **(A)** according to CD location **(B)**, CD behavior **(C)**, CD activity **(D)**, UC extension **(E)** and UC activity **(F)**.

Serum C-reactive protein (CRP) levels were analyzed in the cohort. Patients with CD showed notably higher CRP levels compared to those with UC (**Figure 1A**). Among CD patients, CRP was highest in those patients with colonic location, compared with ileal and ileocolonic ones (**Figure 1B**), and it increased progressively in patients with stricturing and penetrating behavior compared to inflammatory behavior (**Figure 1C**). CRP levels also correlated well with clinical activity indices, rising with disease severity (**Figure 1D**). In UC, CRP was highest extensive patients and lowest in proctitis group (**Figure 1E**), and it similarly increased with disease activity (**Figure 1F**).

### 3.2 Principal Component Analysis

**Figure 2:**
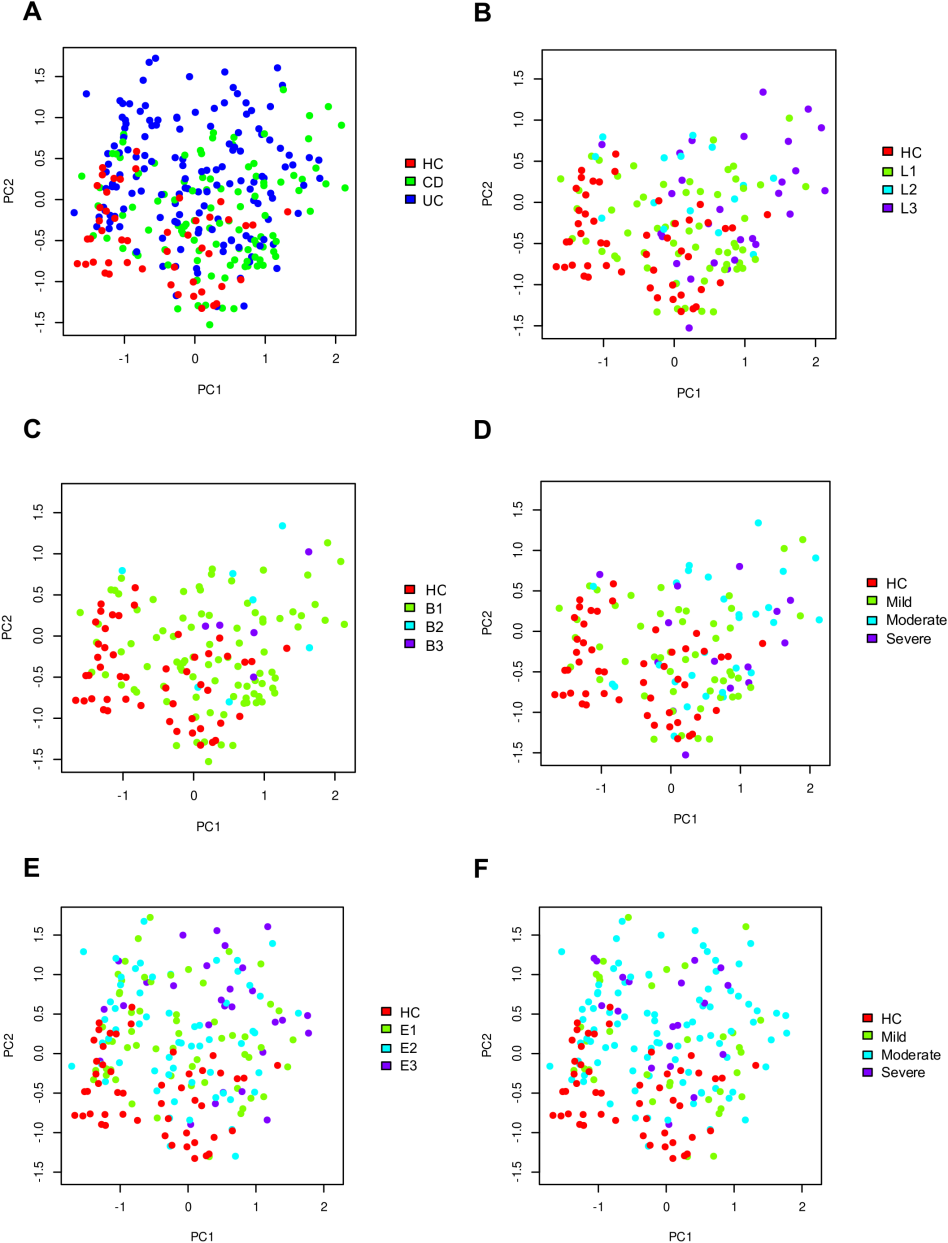
Principal Component Analysis of gut microbiome composition at the phylum level in healthy controls and patients with inflammatory bowel disease (IBD). Panels A-F show the distribution of samples according to: IBD type **(A)**, CD location **(B)**, CD behavior **(C)**, CD severity **(D)**, UC extension **(E)**, and UC severity **(F)**.

The principal component analysis in fecal samples at phylum level revealed distinct clustering patterns. HC samples exhibited tight clustering when compared to IBD patients (**Figure 2A**). In contrast, CD and UC patients showed higher dispersion, with CD samples displaying greater dispersion (**Figure 2A**). Considering CD location, ileal and ileocolonic patients were closely related, while colonic samples formed a distinct cluster (**Figure 2B**). Regarding CD behavior, patients with inflammatory CD clustered tightly, whereas patients with stricturing and penetrating behavior showed the greatest dispersion (**Figure 2C**). Similar trends were observed for CD endoscopic activity, where mild cases clustered more closely than moderate or severe cases (**Figure 2D**). Analysis based on UC extension revealed that patients with proctitis exhibited tighter clustering, while left-sided and extensive colitis patients showed broader dispersion (**Figure 2E**). The pattern of UC activity was comparable to that of CD activity, with mild cases grouped closer together than moderate or severe cases (**Figure 2F**).

### 3.3 Fecal microbiota diversity

**Figure 3:**
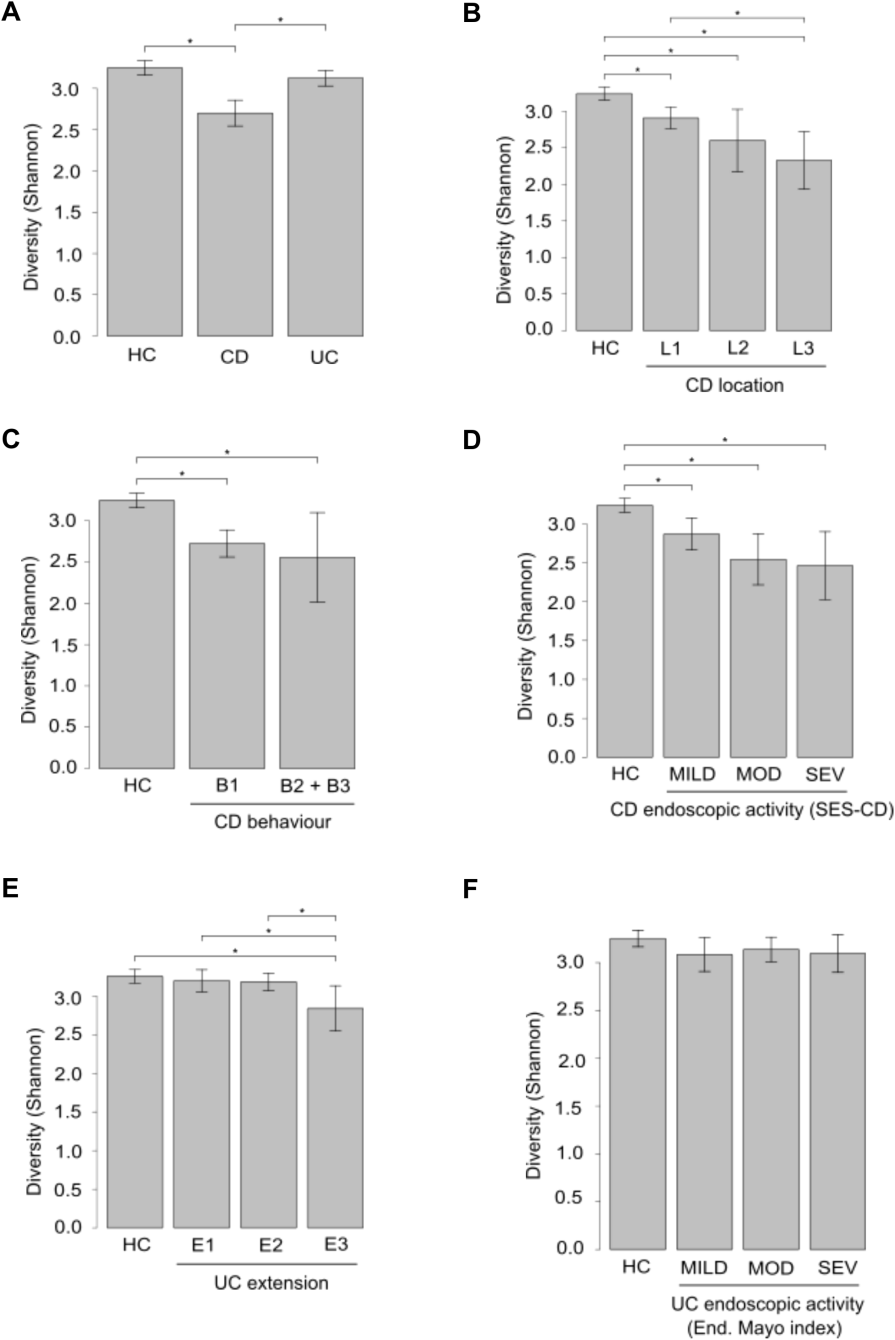
α-Diversity analysis in fecal samples from IBD patients and healthy controls (HC). Panels A-F depict comparisons of α-diversity assessed by the Shannon Diversity Index between HC and patients groups based on: IBD type **(A)**, CD location **(B)**, CD behavior **(C)**, CD severity **(D)**, UC extension **(E)**, and UC severity **(F)**. *p ≤ 0.05.

Analysis of gut microbiota in IBD patients revealed lower α-diversity at species level, with CD patients demonstrating a statistically significant decrease compared to UC patients and HC (**Figure 3A**). Shannon diversity at species level was reduced across CD location and behavior subgroups, compared to HC (**Figures 3B, C**). Furthermore, a negative correlation was observed between endoscopic severity and microbial α-diversity in CD patients (**Figure 3D**). Patients with extensive UC exhibited significantly lower α-diversity compared to patients with left-sided colitis and proctitis and with HC (**Figure 3E**). Microbial α-diversity was not influenced by endoscopic activity in UC patients (**Figure 3F**).

Additionally, β-diversity was calculated using Bray-Curtis dissimilarity to assess the level of species overlap between groups. β-diversity is inversely related to species overlap. Calculation of β-diversity in pair-wise combinations indicated higher overlap in UC vs HC compared to CD vs HC (**Supplementary Figure 1A**). Analysis based on CD location indicated that patients with ileocolonic pattern appeared to have a more distinct microbiota profile than the other studied groups, since dissimilarities between the ileal-ileocolonic combination and colonic-ileocolonic combination were higher than the other pair-wise combinations between CD location groups (**Supplementary Figure 1B**). Considering CD behavior, the combination of stricturing and penetrating behavior vs HC had more β-diversity than the combination of inflammatory behavior vs HC; therefore, stricturing and penetrating behavior exhibited more differences compared to HC than the inflammatory behavior (**Supplementary Figure 1C**). Additionally, β-diversity index increased with disease activity in CD patients (**Supplementary Figure 1D**), whereas no differences in β-diversity were found both between extension groups and between activity groups in UC patients (**Supplementary Figures 1E, 1F**).

### 3.4 Taxonomic characterization of gut microbiota in IBD subgroups

#### 3.4.1 IBD patients vs HC

**Figure 4:**
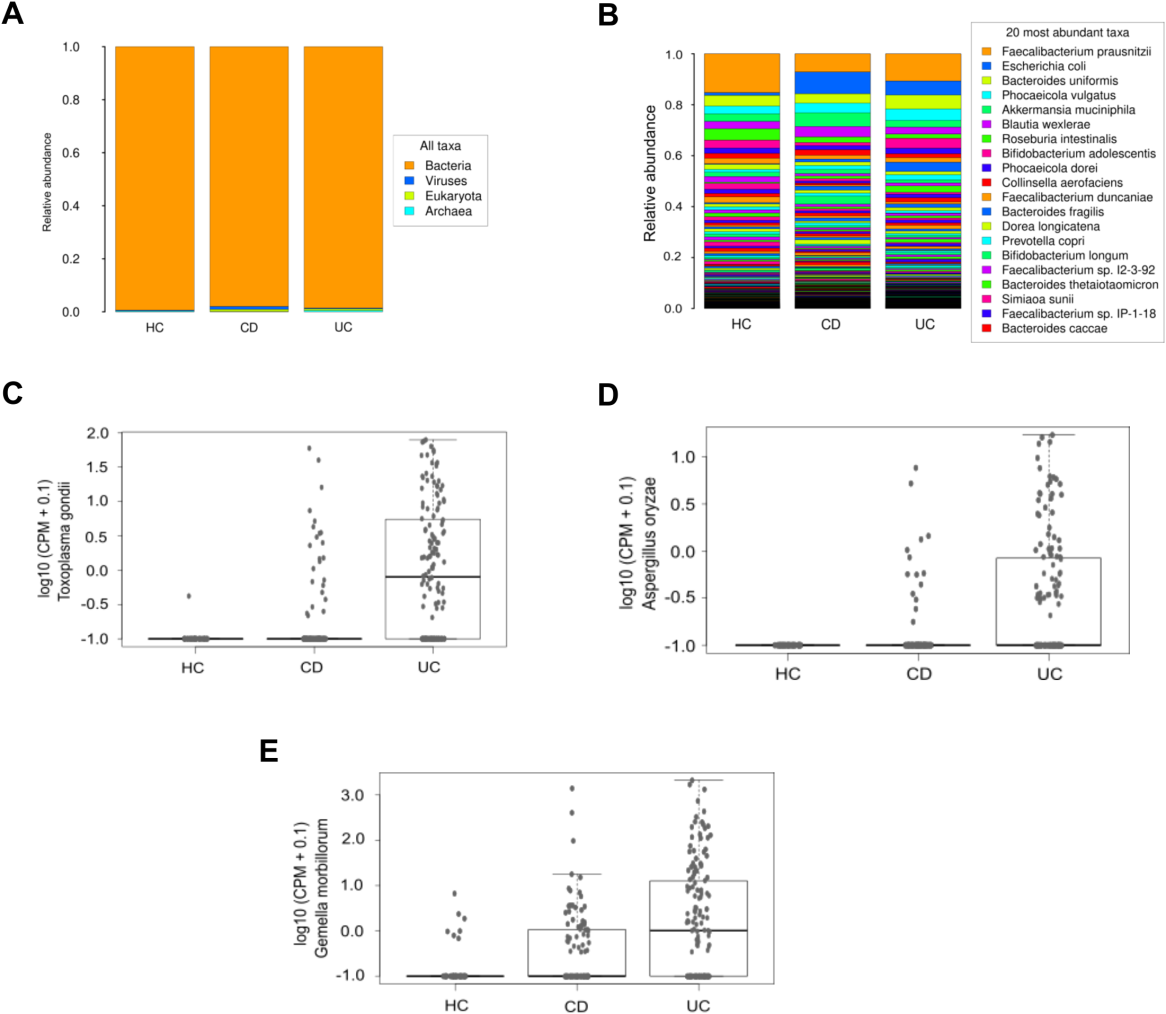
Microbiological composition at domain and species levels in the IBDomics cohort. Histograms exhibit taxonomic distribution at domain **(A)** and species **(B)** levels across the CD, UC and HC study groups. The graphs represent a selection of species with significant differences (Kruskal-Wallis p_value < 0.05) between groups: *Toxoplasma gondii* **(C)**, *Aspergillus oryzae* **(D)**, and *Gemella morbillorum* **(E)** levels, expressed in counts per million.

Disparities in taxonomic profiles were identified among different study groups. At the domain level, CD patients exhibited an increase in relative abundance in virus and eukaryote levels compared to both UC patients and HC (**Figure 4A**). Furthermore, there was a trend towards a higher relative abundance of *Escherichia coli* and a lower relative proportion of *Faecalibacterium prausnitzii* in IBD patients compared to HC (**Figure 4B**). Limma analysis identified the most significant differences in species composition between IBD and UC patients relative to HC, highlighting *Toxoplasma gondii* and *Gemella morbillorum* (**Table 2**). These species were also prominently significant in the Kruskal-Wallis analysis, alongside *Aspergillus oryzae* (**Figures 4C-E**). Notably, CD patients exhibited a decrease in several species within the *Adlercreutzia* genus, and in turn an increase in species of the *Shigella* genus, both compared to HC (**Table 2**).

**Table 2:** Total species detected and species with the most significant differences in IBD patients compared to HC individuals, and between Crohn’s disease and ulcerative colitis patients.

Comparisons	Total species detected	Total species with significant differences	Top six species with significant differences (n-fold)
IBD vs HC	685	260	Toxoplasma gondii (4.53) Gemella morbillorum (3.9) Shigella flexneri (3.7) Streptococcus constellatus (3.6) Streptococcus anginosus (3.4) Escherichia fergusonii (3.3)
CD vs HC	685	363	Adlercreutzia hattorii (-4.8) Phocaeicola coprocola (-4.7) Methanobrevibacter smithii (-4.2) Adlercreutzia equolifaciens (-3.9) Shigella flexneri (3.7) Shigella dysenteriae (3.7)
UC vs HC	685	130	Toxoplasma gondii (5.6) Gemella morbillorum (4.9) Aggregatibacter sp. oral taxon 513 (4.1) Aspergillus oryzae (4.0) Shigella flexneri (3.9) Anaerococcus obesiensis (3.9)
CD vs UC	685	217	Methanobrevibacter smithii (-3.6) Bifidobacterium bifidum (-3.2) Peptoniphilus sp. SAHP1 (-3.1) Enterococcus lactis (3.0) Lactobacillus gasseri (2.8) Lactobacillus paragasseri (2.7)

HC, healthy controls; IBD, inflammatory bowel disease; CD, Crohn´s disease; UC, ulcerative colitis.

Abundance differences are expressed as fold changes (n-fold) between the first and the second conditions being compared, and are significant with adjusted p_value < 0.05, according to tests performed with limma.

#### 3.4.2 CD location

**Figure 5:**
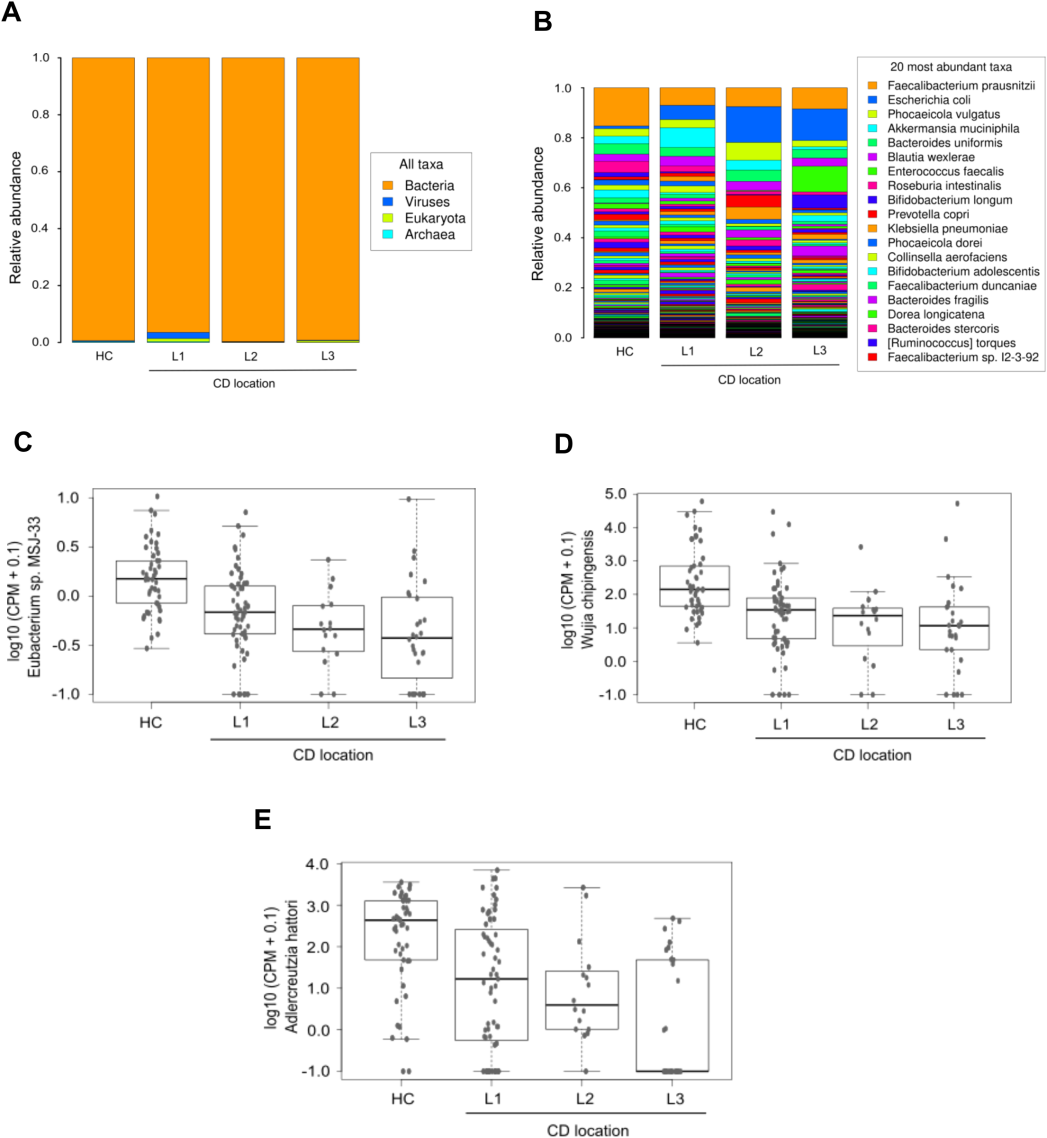
Taxonomic abundance based on Crohn’s disease location. Stacked bars in both histograms show the average relative abundances of all domains **(A)** and the most prevalent species **(B)** identified in the three CD patient groups defined according to disease location, and in HC. Panels C-E exhibit a selection of species with significant differences (Kruskal-Wallis p_value < 0.05) between groups: *Eubacterium* sp. *MSJ-33* **(C)**, *Wijia chipingensis* **(D)**, and *Adlercreutzia hattorii* **(E)** levels, expressed in counts per million.

Differences were observed in domain taxonomic rank among CD patients based on disease location. Patients with colonic and ileocolonic CD shared similar profiles compared to HC. In contrast, ileal CD patients displayed a tendency towards higher relative levels of virus and eukaryotic domains (**Figure 5A**). Additionally, there was a tendency towards increased relative proportion of *Escherichia coli* in all the CD patient groups defined according to disease location, with the highest levels in the colonic group, compared to HC and patients with ileal or ileocolonic location. Conversely, *Faecalibacterium prausnitzii* levels were notably lower across all three patient groups in comparison to HC, and no differences were found between the groups analyzed (**Figure 5B**). Limma analysis underscored a substantial decreasing gradient in the levels of *Phocaeicola coprocola* and various *Adlercreutzia* species between CD location subgroups compared to HC. However, *Shigella flexneri* exhibited an opposing trend (**Table 3**). Kruskal-Wallis testing highlighted *Eubacterium* sp. *MSJ-33*, *Wijia chipingensis*, and *Adlercreutzia hattorii* as significantly diminished species in gradient between CD location groups compared to HC (**Figures 5C-E**).

**Table 3:** Total species detected and species with the most significant differences in Crohn´s disease patients according to disease location.

Comparisons	Total species detected	Total species with significant differences	Top six species with significant differences (n-fold)
L1 vs HC	556	79	Adlercreutzia hattorii (-3.9) Methanobrevibacter smithii (-3.9) Toutatisvirus toutatis (-3.9) Phocaeicola coprocola (-3.7) Bifidobacterium adolescentis (-3.4) Brigitvirus brigit (-3.4)
L2 vs HC	556	26	Shigella flexneri (6.9) Salmonella entérica (5.4) Ruminococcus bicirculans (-4.5) Bifidobacterium adolescentis (-4.4) Streptococcus lutetiensis (4.3) Veillonella sp. S12025-13 (4.3)
L3 vs HC	556	289	Adlercreutzia hattorii (-8.0) Phocaeicola coprocola (-7.1) Adlercreutzia equolifaciens (-7.0) Methanobrevibacter smithii (-5.7) Shigella flexneri (5.1) Alistipes communis (-5.1)
L1 vs L2	556	0	
L1 vs L3	556	102	Proteus mirabilis (-4.2) Adlercreutzia hattorii (4.1) Adlercreutzia equolifaciens (4.1) Aeromonas salmonicida (3.5) Toxoplasma gondii (-3.5) Arachnia rubra (-3.4)
L2 vs L3	556	1	Gemella haemolysans (-3.9)

HC, healthy controls; L1: ileal; L2: colonic; L3: ileocolonic.

Abundance differences are expressed as fold changes (n-fold) between the first and the second conditions being compared, and are significant with adjusted p_value < 0.05, according to tests performed with limma.

#### 3.4.3 CD behavior

**Figure 6:**
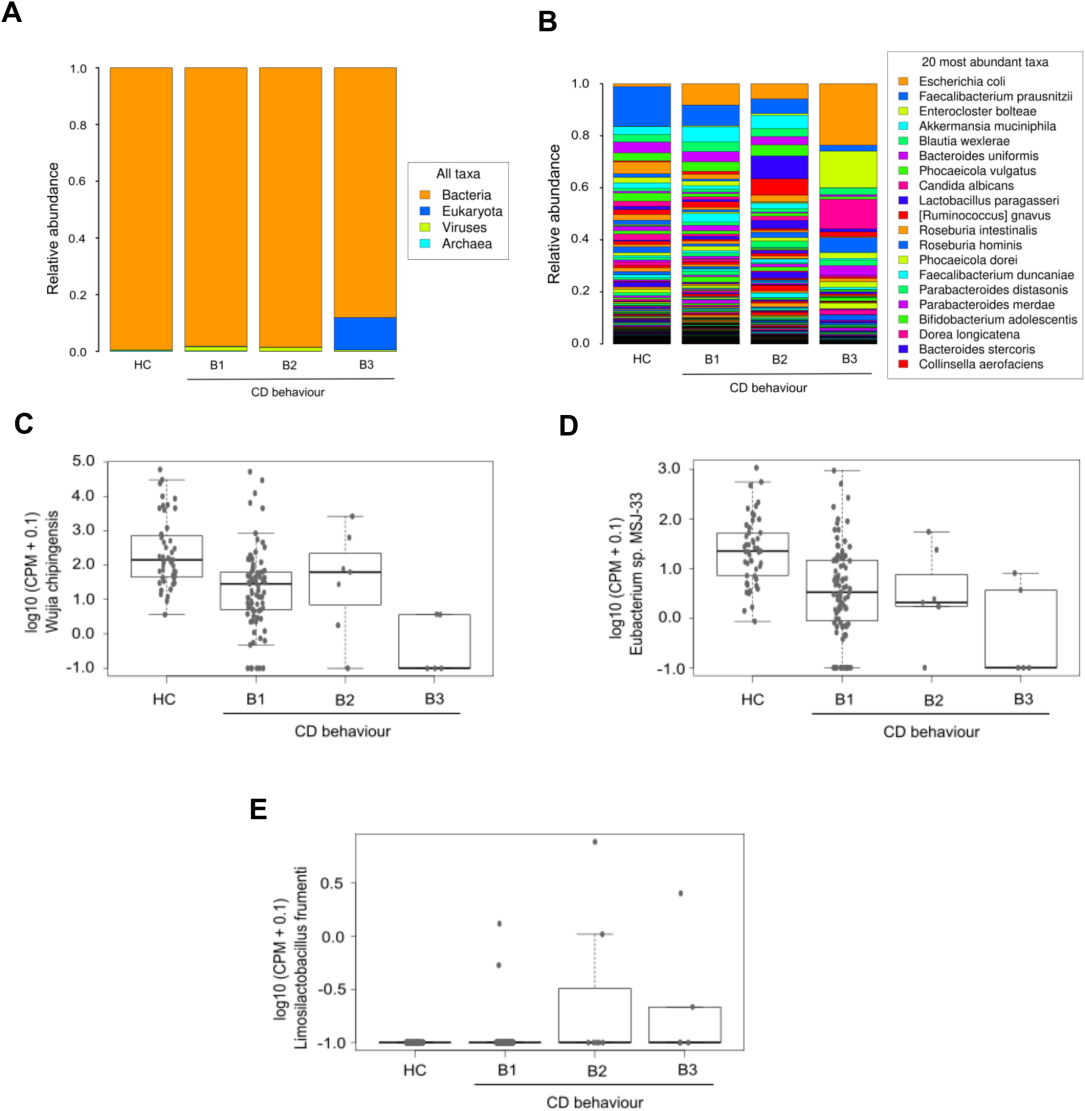
Microbiological profile corresponding to Crohn’s disease behavior. The first two panels display the average relative abundances of all domains **(A)** and the most common species **(B)** in the three CD patient groups defined according to disease behavior, and in HC. The remaining graphics show a selection of species with significant differences (Kruskal-Wallis p_value < 0.05) between groups: *Wijia chipingensis* **(C)**, *Eubacterium* sp. *MSJ-33* **(D)**, and *Limosilactobacillus frumenti* **(E)** levels, represented in counts per million.

Variations in domain taxa were also observed among different CD behavior. Patients with inflammatory and stricturing behavior demonstrated similar profiles, with a slight increase in virus levels. Unlike HC and the other CD behavior groups, patients with penetrating CD showed higher amounts of the eukaryotic domain (**Figure 6A**). Among the ten most abundant species in the penetrating group, *Candida albicans* was the only eukaryotic species identified, while the rest belonged to the bacterial domain. Relative abundance results at the species level showcased increased *Escherichia coli* in all CD behavior groups compared to HC, especially higher in penetrating behavior. In contrast, *Faecalibacterium prausnitzii* decreased in all CD behavior groups, compared to HC (**Figure 6B**). Limma analysis revealed significant decreases in *Phocaeicola coprocola*, *Adlercreutzia hattorii*, and *Methanobrevibacter smithii* among CD behavior groups, alongside a significant increase in *Lactobacillus crispatus* (**Table 4**). Kruskal-Wallis test showed similar findings to those found in the CD location comparison; in addition, this analysis found high levels of *Limosilactobacillus frumenti* in the structuring and penetrating groups (**Figures 6C-E**).

**Table 4:** Total species detected and species with the most significant differences in Crohn’s disease patients according to disease behavior.

Comparisons	Total species detected	Total species with significant differences	Top six species with significant differences (n-fold)
B1 vs HC	556	193	Phocaeicola coprocola (-4.6) Adlercreutzia hattorii (-4.6) Methanobrevibacter smithii (-4.2) Shigella flexneri (4.1) Adlercreutzia equolifaciens (-3.9) Bifidobacterium adolescentis (-3.8)
B2 + B3 vs HC	556	116	Adlercreutzia hattorii (-8.1) Lactobacillus crispatus (7.7) Lactobacillus paragasseri (7.2) Phocaeicola coprocola (-6.8) Alistipes ihumii (-6.4) Methanobrevibacter smithii (-6.3)
B1 vs B2 + B3	556	2	Lactobacillus crispatus (-5.7) Lachnoclostridium sp. YL32 (-3.2)

HC, healthy controls; B1: inflammatory; B2: stricturing; B3: penetrating.

Abundance differences are expressed as fold changes (n-fold) between the first and the second conditions being compared, and are significant with adjusted p_value < 0.05, according to tests performed with limma.

#### 3.4.4 CD activity

**Figure 7:**
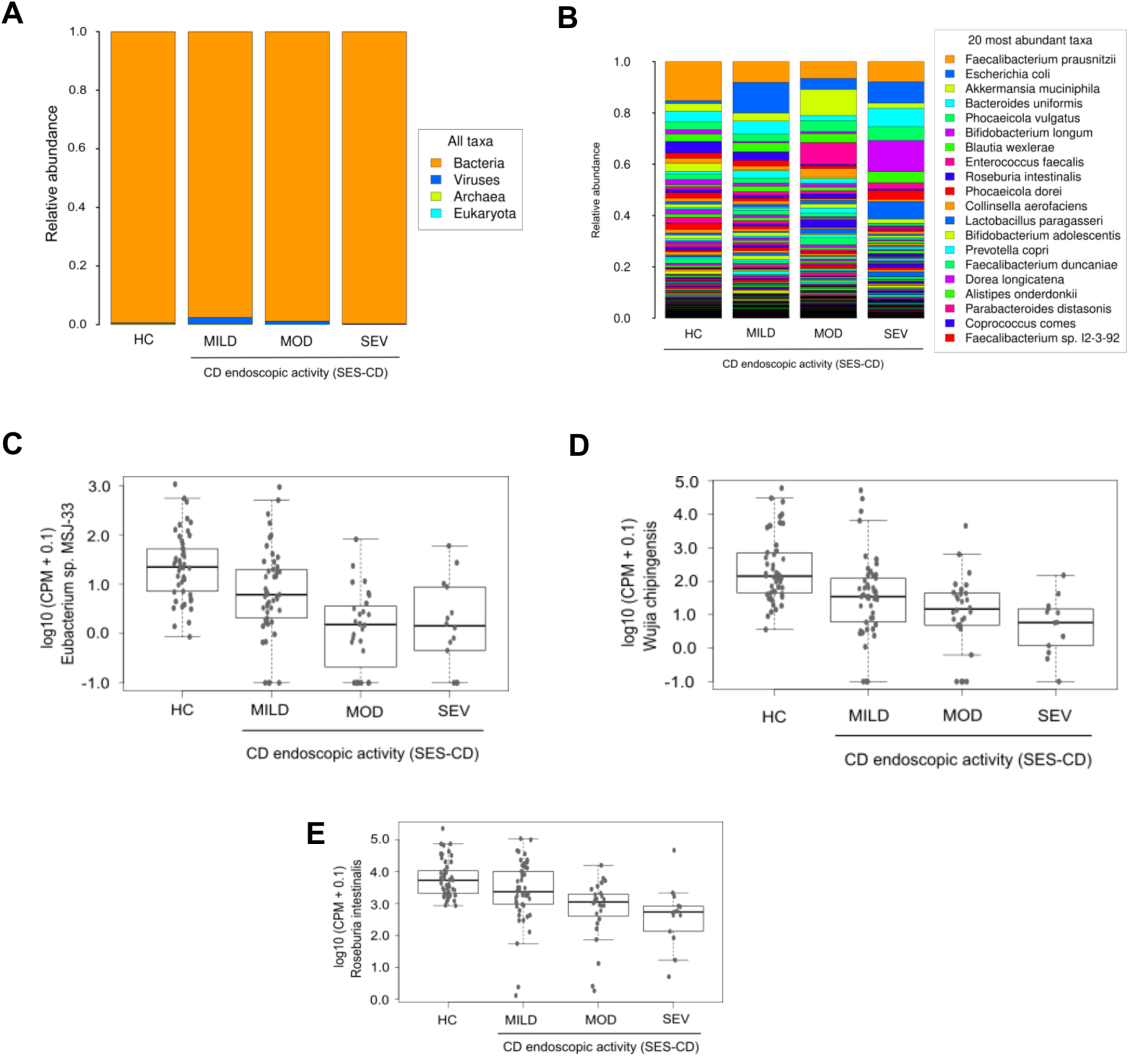
Metagenomics profile in Crohn’s disease patients categorized according to their disease activity. Histograms show the taxonomic abundances of all domains **(A)** and the most common species **(B)**, detected in the three CD patient groups defined according to disease activity, and in HC. Graphs show a selection of species with significant differences (Kruskal-Wallis p_value < 0.05) between groups: *Eubacterium* sp. *MSJ-33* **(C)**, *Wijia chipingensis* **(D)**, and *Roseburia intestinalis* **(E)** levels, represented in counts per million.

Evaluation of taxonomic abundance at the domain level indicated that patients with severe CD exhibited relatively higher bacteria abundance than other groups (**Figure 7A**). Analysis at the species level showed an increase in relative proportion of *Escherichia coli* in the three CD activity groups compared to HC, being higher in the mild group, whereas *Faecalibacterium prausnitzii* showed a decreased relative proportion compared to HC, similar to that of the other activity groups (**Figure 7B**). Limma analysis unveiled a significant reduction in the levels of *Phocaeicola coprocola* and several *Adlercreutzia* genus species in each CD activity group relative to HC. Conversely, the pathogen *Shigella flexneri* was significantly increased in all three CD activity groups compared to HC (**Table 5**). The most significant species identified by the Kruskal-Wallis test included *Eubacterium* sp. *MSJ-33*, *Wijia chipingensis* and *Roseburia intestinalis*, all exhibiting direct decrease in CD activity groups compared to HC (**Figures 7C-E**).

**Table 5:** Total species detected and species with the most significant differences in Crohn´s disease patients according to endoscopic severity.

Comparisons	Total species detected	Total species with significant differences	Top six species with significant differences (n-fold)
Mild vs HC	546	16	Phocaeicola coprocola (-4.6) Shigella flexneri (3.8) Adlercreutzia hattorii (-3.7) Salmonella entérica (3.5) Shigella dysenteriae (3.5) Shigella sonnei (3.4)
Moderate vs HC	546	268	Adlercreutzia hattorii (-6.4) Streptococcus anginosus (5.8) Methanobrevibacter smithii (-5.7) Adlercreutzia equolifaciens (-5.4) Ruminococcus bicirculans (-5.0) Bifidobacterium pseudocatenulatum (-5.0)
Severe vs HC	546	88	Lactobacillus paragasseri (6.8) Phocaeicola coprocola (-6.6) Lactobacillus gasseri (6.5) Shigella flexneri (6.0) Adlercreutzia hattorii (-6.0) Adlercreutzia equolifaciens (-5.6)
Severe vs Mild	546	0	
Severe vs Moderate	546	0	

HC, healthy controls.

Abundance differences are expressed as fold changes (n-fold) between the first and the second conditions being compared, and are significant with adjusted p_value < 0.05, according to tests performed with limma.

#### 3.4.5 UC extension

Examination of relative abundance histograms at the domain level revealed a slight and direct increase in virus levels across all three UC groups (proctitis, left-sided colitis and extensive colitis), with the highest levels detected in extensive colitis (**Supplementary Figure 2A**). At the species level *Escherichia coli* levels showed a gradual increase across UC subgroups compared to HC, while *Faecalibacterium prausnitzii* exhibited an opposite trend. Notably, *Bacteroides uniformis* was one of the most prevalent species without any variations across the UC extension groups (**Supplementary Figure 2B**). Limma analysis highlighted significant increases in *Toxoplasma gondii, Aggregatibacter* sp. *oral taxon 513*, and *Gemella morbillorum* across all three UC groups relative to HC. However, several species within the *Adlercreutzia* and *Alistipes* genera showed significant decreases in the extensive colitis group compared to HC. Additionally, these genera and certain Alistipes species were notably decreased in patients with extensive colitis alone and combined with left-sided colitis compared to the proctitis group (**Table 6**). The Kruskal-Wallis test confirmed the significance of *Toxoplasma gondii, Aspergillus oryzae* and *Neisseria macacae*, with higher levels of the former two in the left-sided colitis group and elevated *Neisseria macacae* levels in the extensive colitis group (**Supplementary Figures 2C-E**).

**Table 6:** Total species detected and species with the most significant differences in ulcerative colitis patients according to disease extension.

Comparisons	Total species detected	Total species with significant differences	Top six species with significant differences (n-fold)
E1 vs HC	606	19	Gemella morbillorum (5.4) Toxoplasma gondii (4.6) Anaerococcus obesiensis (4.2) Aggregatibacter sp. oral taxon 513 (3.6) Dialister pneumosintes (3.5) Streptococcus constellatus (3.3)
E2 vs HC	606	23	Toxoplasma gondii (6.1) Gemella morbillorum (4.4) Aspergillus oryzae (3.9) Shigella flexneri (3.8) Streptococcus constellatus (3.3) Streptococcus anginosus (3.2)
E3 vs HC	606	218	Toxoplasma gondii (6.2) Streptococcus constellatus (5.8) Adlercreutzia hattori (-5.6) Aggregatibacter sp. oral taxon 513 (5.5) Adlercreutzia equolifaciens (-5.4) Fusobacterium nucleatum (5.3)
E1 vs E2	606	0	
E1 vs E3	606	132	Adlercreutzia equolifaciens (5.1) Alistipes indistinctus (4.7) Veillonella nakazawae (-4.6) Adlercreutzia hattorii (4.6) Alistipes communis (4.6) Alistipes shahii (4.4)
E1 + E2 vs E3	580	107	Alistipes communis (4.6) Adlercreutzia equolifaciens (4.4) Shigella boydii (-4.4) Alistipes indistinctus (4.3) Alistipes onderdonkii (4.3) Adlercreutzia hattorii (4.0)

HC, healthy controls; E1: proctitis; E2: Left sided; E3: Extensive.

Abundance differences are expressed as fold changes (n-fold) between the first and the second conditions being compared, and are significant with adjusted p_value < 0.05, according to tests performed with limma.

#### 3.4.6 UC activity

Evaluation of domain-level taxonomic abundance showed the highest virus levels in patients with moderate UC and elevated eukaryotic taxa in severe UC patients (**Supplementary Figure 3A**). At the species level, UC patients exhibited slightly lower relative proportions of *Faecalibacterium prausnitzii* compared to HC, while *Escherichia coli* showed higher relative levels in all the groups compared to HC. Additionally, *Phocaeicola coprocola* and *Bacteroides uniformis* emerged as the second and third most abundant species, respectively. Notably, *Phocaeicola coprocola* levels were relatively higher in severe UC patients compared to other groups, whereas *Bacteroides uniformis* levels remained consistent among the studied groups (**Supplementary Figure 3B**). Limma analysis demonstrated a direct relationship between the severity of UC activity and *Toxoplasma gondii* and *Aspergillus oryzae* levels. Conversely, *Gemella morbillorum* levels displayed an inverse correlation (**Table 7**). These species were identified as the most significant in the Kruskal-Wallis test and exhibited similar trends among the study groups as observed in the limma analysis (**Supplementary Figures 3C-E**).

**Table 7:** Total species detected and species with the most significant differences in ulcerative colitis patients according to endoscopic severity.

Comparisons	Total species detected	Total species with significant differences	Top six species with significant differences (n-fold)
Mild vs HC	606	24	Gemella morbillorum (5.8) Dialister pneumosintes (4.8) Toxoplasma gondii (4.6) Shigella flexneri (3.8) Anaerococcus obesiensis (3.7) Bacteroides sp. ZJ-18 (3.6)
Moderate vs HC	606	85	Toxoplasma gondii (5.6) Gemella morbillorum (4.8) Streptococcus constellatus (4.7) Aggregatibacter sp. oral taxon 513 (4.0) Streptococcus anginosus (3.6) Aspergillus oryzae (3.6)
Severe vs HC	606	3	Toxoplasma gondii (6.9) Aspergillus oryzae (4.5) Pseudobutyrivibrio xylanivorans (-3.3)
Severe vs Mild	606	0	
Severe vs Moderate	606	0	

HC, healthy controls.

Abundance differences are expressed as fold changes (n-fold) between the first and the second conditions being compared, and are significant with adjusted p_value < 0.05, according to tests performed with limma.

### 3.5 Functional analyses

Analysis with HUMAnN3 detected 256 pathways that differed significantly between HC and IBD patients. The pathways more upregulated in IBD patients compared to HC were glucose and xylose degradation, pyrimidine deoxyribonucleoside degradation, gluconeogenesis, and fatty acid beta-oxidation. Regarding CD patients, 272 pathways were changed, highlighting that thiamine diphosphate production was reduced and nucleotide metabolism was upregulated compared to HC. UC patients displayed 215 modified pathways, showing higher activity of gluconeogenesis and fatty acid metabolism compared to HC (**Supplementary Table 2**). In particular, ileal CD patients exhibit increased nucleotide metabolism activity compared to HC, whereas colonic and ileocolonic CD patients displayed a decrease in lysine and thiamine diphosphate biosynthesis activity compared to HC (**Supplementary Table 3**). Stricturing and penetrating CD groups exhibited significant reductions in amino acid biosynthesis and urea cycle activity compared to HC (**Supplementary Table 4**). CD endoscopic activity also influenced metabolic shifts, increasing the metabolism of carbohydrates and nucleotides; however, there were no significant variations between patients with severe CD compared to mild and moderate CD patients (**Supplementary Table 5**). Regarding UC extension, metabolic alterations were more noticeable in extensive colitis compared to HC, especially showing an increase in glucose metabolism and amino acid biosynthesis (**Supplementary Table 6**). Finally, UC activity was mainly associated with an increase in glucose metabolism and rubisco shunt; however, there were no significant changes between patients with severe CD compared to mild and moderate CD groups (**Supplementary Table 7**).

## 4 Discussion

In this article, we present a comprehensive fecal microbiota study in a large cohort of newly diagnosed, treatment-naïve CD and UC patients, compared to HC. We applied shotgun metagenomics to examine all the microbiological domains present in stool samples from IBD patients and HC, since not only bacteria can promote dysbiosis in IBD (Norman et al., 2015; Sokol et al., 2017; Ghavami et al., 2018). In addition, we analyzed the metagenomics data of the samples considering, in comparison to HC, all the descriptive clinical characteristics of IBD: location and behavior of CD, extent of UC, and severity of both diseases.

Serum C-reactive protein (CRP) is a well-established systemic marker of inflammation widely used in inflammatory bowel disease (IBD) management. CRP is an acute-phase protein produced by the liver in response to pro-inflammatory cytokines such as IL-6, reflecting systemic inflammatory activity (Luan and Yao, 2018). Elevated CRP levels correlate with disease activity in both CD and UC and are commonly used to monitor treatment response (Wagatsuma et al., 2021; Sakurai and Saruta, 2023). Although fecal calprotectin and serum C-reactive protein are valuable non-invasive biomarkers for assessing intestinal inflammation and monitoring disease activity, they serve as complementary tools but cannot substitute for endoscopy.

Firstly, we have shown that naïve IBD patients exhibit different microbial profiles than HC, with bigger difference in CD than in UC. In addition, we found differences in these patients according to their disease characteristics. In terms of abundance, beneficial species were decreased and harmful ones were increased in IBD patients compared to HC. Moreover, we identified species that were less abundant under baseline conditions and varied significantly and noticeably in other pathological states.

Shannon index and Bray-Curtis dissimilarity revealed that CD patients have lower microbial diversity and more different microbial profile than UC compared to HC. However, the microbiota of UC patients is closer to that of HC according to both indexes. These findings indicate a higher degree of dysbiosis in CD patients compared to those with UC, which is in accordance with other studies (Pascal et al., 2017; Serrano-Gómez et al., 2021; Kang et al., 2023).

Previous studies have shown that the gut microbiota of IBD patients is characterized by reduced diversity, decreased abundance of the *Firmicutes* phylum and an increase in the *Proteobacteria* phylum (Frank et al., 2007; Nishino et al., 2018). In our work, we observed a relative reduction in *Faecalibacterium prausnitzi* in IBD patients compared to HC. This Gram-positive bacterium is generally considered an indicator of a healthy gut, and is found in low quantities in many pathologies including IBD (Miquel et al., 2013; Parsaei et al., 2021). Notably, in our study CD patients exhibited the lowest abundance of this bacterium compared to HC and UC. Conversely, *Escherichia coli*, a gammaproteobacteria, was increased in our IBD patients, in accordance with previous studies that have consistently shown increased abundance in IBD patients with higher prevalence in CD than in UC patients (Rhodes, 2007; Schirmer et al., 2019).

Regarding other less abundant species in the microbiota, taxonomic abundance analyses revealed differences between CD and UC, as the species showing the most significant differences compared to HC vary between both groups. The highest differences were observed for the *Adlercreutzia* and *Shigella* genera in CD patients and for *Gemella morbillorum* and *Toxoplasma gondii* in UC patients. We identified two species from the *Adlercreutzia* genus (*Adlercreutzia hattorii* and *Adlercreutzia equolifaciens)* whose abundance decreased most significantly in IBD patients compared to HC. *Adlercreutzia hattorii* was recently discovered in human feces (Sakamoto et al., 2021) and its role in IBD is currently unknown. *Adlercreutzia equolifaciens* acts similarly to *Faecalibacterium prausnitzii*, although its abundance is lower. Galipeau et al. described a reduction of this species in UC patients associated with an inflammatory state (Galipeau et al., 2021).

Furthermore, pathogen species of the *Shigella* genus are known to cause inflammatory destruction of the intestinal epithelial barrier (Kotloff et al., 1999; Phalipon and Sansonetti, 2003). In this respect, previous studies have reported elevated levels of the *Shigella flexneri* pathogen in preclinical models of UC (Ma et al., 2023). Finally, *Gemella morbillorum* has been found to be increased in both CD patients (Yu et al., 2022) and in those with colorectal cancer (Ternes et al., 2020).

In the present study we have identified two species of the *Bacteroidaceae* family that are altered in IBD patients: *Bacteroides uniformis* and *Phocaeicola coprocola*. Although the information about these two species in relation to IBD is limited, the association of other species of this family with IBD has been described. A correlation has been found between a reduction in *Bacteroides* spp. in UC patients and diarrhea and rectal bleeding (Hellmann et al., 2023) and between *Phocaeicola vulgatus* and the presence of active inflammation in CD patients (Gonzalez et al., 2022).

When the CD cohort was divided in groups based on disease characteristics, patients with the lowest α-diversity were those with ileocolonic localization, penetrating behavior, and severe activity, suggesting that lower α-diversity is associated with a more severe manifestation of the disease. However, among UC patients, only those with a pattern of extensive disease showed differences in α-diversity, suggesting that the UC ecosystem is less affected by microbiota diversity than CD, and that from the point of view of fecal microbiota, UC is a milder disease. In relation to these findings, Imhann et al. discovered that α-diversity in patients with UC and colonic CD did not differ from that of HC, whereas this parameter was reduced in patients with ileocolonic and ileal CD (Imhann et al., 2018).

In addition, according to disease location, CD patients with ileocolonic location exhibited the most different microbial profile compared to patients with ileal or colonic location, probably due to the manifestation of the disease in two different organs of the digestive tract. Concerning CD behavior, we found that microbiota in patients with inflammatory pattern is closer to that of HC, while patients with stricturing or penetrating behavior, showed more variation in species. These β-diversity results, along with the α-diversity results revealing that species richness decreased in the stricturing and penetrating behaviors, may indicate that these patients present a worse clinical condition. A similar situation might occur when we categorize these patients based on CD activity. The Bray-Curtis dissimilarity analysis between CD activity subgroups found the highest diversity of species in moderate and severe patterns, being higher in the latest group. A previous study showed similar results, indicating that CD patients had a higher abundance of inflammation-causing microorganisms (Kang et al., 2023). In UC patients, no differences were found between comparisons based on extension and activity, suggesting that species variation is not dependent on a specific UC classification.

Considering taxonomic abundance, we observed a higher abundance of *Escherichia coli* in CD patients with a penetrating behavior, in those with colonic and ileocolonic location, and in UC patients with a pattern of extensive disease. This could suggest a direct increase associated with specific colonic sites of damage. On the other hand, we observed a significant decrease in *Methanobrevibacter smithii* in CD patients, a bacterial species previously found in low proportions in IBD patients (Ghavami et al., 2018) associated with ileal and ileocolonic CD locations. This is the most abundant microbe in the colon capable of producing methane and short-chain fatty acids (Ghoshal et al., 2016; Ghavami et al., 2018). Other species, such as the facultative anaerobic Gram-positive bacteria *Lactobacillus crispatus* and *Lactobacillus* spp., showed a reduction in active UC patients (Fabia et al., 1993). Interestingly, in our study, we detected a significant increase in these bacteria in CD patients with stricturing or penetrating behavior compared to HC. In a preclinical model of colitis induced by DSS administration, the presence of a strain of *Lactobacillus crispatus* aggravated intestinal inflammation, promoting cell infiltration and activating the expression of proinflammatory cytokines such as IL-1β, IL-6, and TNF-α (Zhou et al., 2012), thereby indicating that not all species within this genus may be beneficial. *Roseburia intestinalis* is another beneficial bacterium that decreases in UC patients (Nie et al., 2021). Its metabolite butyrate has been reported to inhibit colon inflammation in a DSS-induced colitis model (Ruan et al., 2022). Another preclinical study showed that administration of this microbe with *Faecalibacterium prausnitzii* and *Bacteroides faecis* significantly alleviated DSS-induced colitis by modulating immune pathways and balancing Treg and Th17 cells (Mohebali et al., 2023). In our work, we found lower levels in CD patients, which varied significantly according to disease activity, with lower levels in patients with higher severity.

The use of shotgun metagenomics has allowed us to identify other types of microbes in certain IBD conditions, along with specific archaea and bacteria. The microorganism showing the most substantial change in our study was *Toxoplasma gondii*. It was increased in multiple comparisons with HC involving IBD patients and patient groups with different characteristics. This protist has been involved in exacerbated inflammation associated with colitis (Saraav et al., 2021). In our cohort, it was primarily elevated in patients with UC, and the extent of this increase was directly related with the extent and severity of the condition, confirming previous findings (Saraav et al., 2021). In these patients, we also observed a significant increase in *Aspergillus oryzae*, especially in those with left-sided extension, moderate, and severe activity. Previous reports are in agreement with this result. It has been previously reported that species of this fungal genus cause intestinal bleeding and infections in immunocompromised UC patients (Marti-Aguado et al., 2017). Additionally, a metagenomic study of biopsies from UC patients showed a significant increase in this genus compared to HC (Qiu et al., 2017). Moreover, *Aspergillus oryzae* is widely used in fermenting traditional Asian foods like soy sauce, miso, and sake (Barbesgaard et al., 1992), and its presence in the gut may reflect intake of fermented products. Such foods can transiently alter gut mycobiome composition (Nomura et al., 2022). Although we lacked detailed dietary data, the increased *Aspergillus oryzae* abundance may stem from consuming these products. Some studies also suggest *Aspergillus oryzae* metabolites have immunomodulatory effects (Nomura et al., 2022; Seidler et al., 2024), potentially relevant in IBD. Further research with dietary assessments is needed to determine if *Aspergillus oryzae* is a transient dietary component or contributes to disease.

On the other hand, the information regarding the association of certain bacterial species detected in our study with IBD is limited. We found a significant abundance of *Aggregatibacter* sp. *oral taxon 513* and *Neisseria macacae* in UC patients. Although some species of these genera have been associated with the oral cavity and linked to periodontal diseases (Haubek, 2010; Weyand, 2017), only one study has reported species of the *Aggregatibacter* genus in the stool of IBD patients (Kitamoto et al., 2020). Regarding to the *Neisseria* genus, it is less abundant in the salivary microbiota of IBD patients compared to that of HC (Said et al., 2014). Hirano et al. observed a decrease in the abundance of this genus in intestinal mucosa biopsies from UC patients compared to HC, suggesting a potential role in maintaining microbial homeostasis (Hirano et al., 2018). However, in our cohort, *Neisseria macacae* is more abundant in patients with extensive UC compared to other IBD patients and HC. Given this contradictory evidence, further studies are needed to define more precisely the role of this genus in IBD. *Alistipes finegoldii*, a member of the relatively new genus *Alistipes* (Rautio et al., 2003), could exert a protective effect against colitis because its levels decrease in mice with this condition (Parker et al., 2020). This genus, along with other anaerobic bacteria, show a notable reduction in CD patients when compared to HC (Amos et al., 2021). In our study, we found a decrease in several species of this genus in both CD and UC patients, confirming the aforementioned findings.

This work has several limitations. Although the shotgun technology can detect bacteria, eukaryotes, archaea and viruses (Jovel et al., 2016), we found that viruses were barely detected. This could be because viruses are present in very small quantities during the bioinformatics analysis and metagenomics tools tend to underestimate them (Rampelli et al., 2016). Moreover, although this study includes a large patient cohort, exceeding that of other metagenomics studies, further research is needed to validate these findings. Additionally, we did not collect detailed dietary information from participants, which limits our ability to assess the potential influence of recent dietary intake-particularly of fermented foods-on the presence and abundance of certain microbial taxa, such as Aspergillus oryzae, in the gut microbiota.

This study has several strengths. First, the sample size of our patient cohort is large, whereas most previous metagenomics studies have involved smaller groups. Second, our study includes newly diagnosed patients who had not yet received any treatment for IBD and were therefore free of the confounding effect of treatment on the microbiota. Third, we have been able to identify several species that have not been previously documented in the literature, and whose role in IBD remains unknown.

In conclusion, this study shows that the microbial ecosystem is more altered in patients with CD than in those with UC, and that the change is bigger in those with stenosing and fistulizing behavior at the time of diagnosis, supporting an association between CD disease severity and changes in the gut microbiota. Moreover, we have documented several new microbial species, including some whose involvement in IBD has not been previously described. This is a landmark study that describes the gut microbiota with a special methodology in a very special cohort of newly diagnosed untreated patients, thereby providing the basis for future studies to analyze the relationship between the gut microbiota and the host.

## Data Availability

The datasets presented in this study can be found in online repositories. The names of the repository/repositories and accession number(s) can be found below: https://www.ncbi.nlm.nih.gov/sra/PRJNA1198911, PRJNA1198911.

## References

[B1] Adamina M. Bonovas S. Raine T. Spinelli A. Warusavitarne J. Armuzzi A.. (2020). ECCO guidelines on therapeutics in crohn’s disease: surgical treatment. J. Crohns Colitis 14, 155–168. doi: 10.1093/ecco-jcc/jjz187 31742338

[B2] Aldars-garcía L. Marin A. C. Chaparro M. Gisbert J. P. (2021). The interplay between immune system and microbiota in inflammatory bowel disease: A narrative review. Int. J. Mol. Sci. 22, 1–15. doi: 10.3390/ijms22063076 PMC8002696 33802883

[B3] Altomare A. Putignani L. Del Chierico F. Cocca S. Angeletti S. Ciccozzi M.. (2019). Gut mucosal-associated microbiota better discloses inflammatory bowel disease differential patterns than faecal microbiota. Digestive Liver Dis. 51, 648–656. doi: 10.1016/j.dld.2018.11.021 30573380

[B4] Amos G. C. A. Sergaki C. Logan A. Iriarte R. Bannaga A. Chandrapalan S.. (2021). Exploring how microbiome signatures change across inflammatory bowel disease conditions and disease locations. Sci. Rep. 11, 1–19. doi: 10.1038/s41598-021-96942-z 34548500 PMC8455643

[B5] Andrews S. (2010). FastQC - A quality control tool for high throughput sequence data (Babraham Institute, Babraham, Cambridgeshire, United Kingdom: Babraham Bioinformatics). Available at: http://www.bioinformatics.babraham.ac.uk/projects/fastqc/ (Accessed May 11, 2022).

[B6] Barbesgaard P. Heldt-Hansen H. P. Diderichsen B. (1992). On the safety of Aspergillus oryzae: a review. Appl. Microbiol. Biotechnol. 36, 569–572. doi: 10.1007/BF00183230 1368061

[B7] Bray J. R. Curtis J. T. (1957). An ordination of the upland forest communities of southern Wisconsin. Ecol. Monogr. 27, 325–349. doi: 10.2307/1942268

[B8] Doherty M. K. Ding T. Koumpouras C. Telesco S. E. Monast C. Das A.. (2018). Fecal microbiota signatures are associated with response to ustekinumab therapy among crohn’s disease patients. mBio 9, e02120-17. doi: 10.1128/mBio.02120-17 PMC5850325 29535202

[B9] Dovrolis N. Michalopoulos G. Theodoropoulos G. E. Arvanitidis K. Kolios G. Sechi L. A.. (2020). The interplay between mucosal microbiota composition and host gene-expression is linked with infliximab response in inflammatory bowel diseases. Microorganisms 8, 1–19. doi: 10.3390/microorganisms8030438 PMC7143962 32244928

[B10] Elhag D. A. Kumar M. Saadaoui M. Akobeng A. K. Al-Mudahka F. Elawad M.. (2022). Inflammatory bowel disease treatments and predictive biomarkers of therapeutic response. Int. J. Mol. Sci. 23, 1–25. doi: 10.3390/ijms23136966 PMC9266456 35805965

[B11] Fabia R. Ar’Rajab A. Johansson M. L. Andersson R. Willén R. Jeppsson B.. (1993). Impairment of bacterial flora in human ulcerative colitis and experimental colitis in the rat. Digestion 54, 248–255. doi: 10.1159/000201045 8243838

[B12] Fernández-Tomé S. Moreno L. O. Chaparro M. Gisbert J. P. (2021). Gut microbiota and dietary factors as modulators of the mucus layer in inflammatory bowel disease. Int. J. Mol. Sci. 22, 1–24. doi: 10.3390/ijms221910224 PMC8508624 34638564

[B13] Frank D. N. St. Amand A. L. Feldman R. A. Boedeker E. C. Harpaz N. Pace N. R. (2007). Molecular-phylogenetic characterization of microbial community imbalances in human inflammatory bowel diseases. Proc. Natl. Acad. Sci. U.S.A. 104, 13780–13785. doi: 10.1073/pnas.0706625104 17699621 PMC1959459

[B14] Franzosa E. A. Hsu T. Sirota-Madi A. Shafquat A. Abu-Ali G. Morgan X. C.. (2015). Sequencing and beyond: Integrating molecular “omics” for microbial community profiling. Nat. Rev. Microbiol. 13, 360–372. doi: 10.1038/nrmicro3451 25915636 PMC4800835

[B15] Franzosa E. A. McIver L. J. Rahnavard G. Thompson L. R. Schirmer M. Weingart G.. (2018). Species-level functional profiling of metagenomes and metatranscriptomes. Nat. Methods 15, 962–968. doi: 10.1038/s41592-018-0176-y 30377376 PMC6235447

[B16] Galipeau H. J. Caminero A. Turpin W. Bermudez-Brito M. Santiago A. Libertucci J.. (2021). Novel fecal biomarkers that precede clinical diagnosis of ulcerative colitis. Gastroenterology 160, 1532–1545. doi: 10.1053/j.gastro.2020.12.004 33310084

[B17] Ghavami S. B. Rostami E. Sephay A. A. Shahrokh S. Balaii H. Aghdaei H. A.. (2018). Alterations of the human gut Methanobrevibacter smithii as a biomarker for inflammatory bowel diseases. Microb. Pathog. 117, 285–289. doi: 10.1016/j.micpath.2018.01.029 29477743

[B18] Ghoshal U. Shukla R. Srivastava D. Ghoshal U. C. (2016). Irritable bowel syndrome, particularly the constipation-predominant form, involves an increase in Methanobrevibacter smithii, which is associated with higher methane production. Gut Liver 10, 932–938. doi: 10.5009/gnl15588 27458176 PMC5087933

[B19] Gonzalez C. G. Mills R. H. Zhu Q. Sauceda C. Knight R. Dulai P. S.. (2022). Location-specific signatures of Crohn’s disease at a multi-omics scale. Microbiome 10, 1–15. doi: 10.1186/s40168-022-01331-x 35999575 PMC9400277

[B20] Guo X. Huang C. Xu J. Xu H. Liu L. Zhao H.. (2022). Gut microbiota is a potential biomarker in inflammatory bowel disease. Front. Nutr. 8. doi: 10.3389/fnut.2021.818902 PMC8814525 35127797

[B21] Haubek D. (2010). The highly leukotoxic JP2 clone of Aggregatibacter actinomycetemcomitans: Evolutionary aspects, epidemiology and etiological role in aggressive periodontitis. APMIS 118, 1–53. doi: 10.1111/j.1600-0463.2010.02665.x 21214629

[B22] Hellmann J. Ta A. Ollberding N. J. Bezold R. Lake K. Jackson K.. (2023). Patient-reported outcomes correlate with microbial community composition independent of mucosal inflammation in pediatric inflammatory bowel disease. Inflammation Bowel Dis. 29, 286–296. doi: 10.1093/ibd/izac175 PMC9890220 35972440

[B23] Hirano A. Umeno J. Okamoto Y. Shibata H. Ogura Y. Moriyama T.. (2018). Comparison of the microbial community structure between inflamed and non-inflamed sites in patients with ulcerative colitis. J. Gastroenterol. Hepatol. (Australia) 33, 1590–1597. doi: 10.1111/jgh.14129 29462845

[B24] Hou K. Wu Z. X. Chen X. Y. Wang J. Q. Zhang D. Xiao C.. (2022). Microbiota in health and diseases. Signal Transduct Target Ther. 7, 1–28. doi: 10.1038/s41392-022-00974-4 35461318 PMC9034083

[B25] Imhann F. Vich Vila A. Bonder M. J. Fu J. Gevers D. Visschedijk M. C.. (2018). Interplay of host genetics and gut microbiota underlying the onset and clinical presentation of inflammatory bowel disease. Gut 67, 108–119. doi: 10.1136/gutjnl-2016-312135 27802154 PMC5699972

[B26] Jovel J. Patterson J. Wang W. Hotte N. O’Keefe S. Mitchel T.. (2016). Characterization of the gut microbiome using 16S or shotgun metagenomics. Front. Microbiol. 7. doi: 10.3389/fmicb.2016.00459 PMC4837688 27148170

[B27] Kang D. Y. Park J. L. Yeo M. K. Kang S. B. Kim J. M. Kim J. S.. (2023). Diagnosis of Crohn’s disease and ulcerative colitis using the microbiome. BMC Microbiol. 23, 1–14. doi: 10.1186/s12866-023-03084-5 37951857 PMC10640746

[B28] Kitamoto S. Nagao-Kitamoto H. Hein R. Schmidt T. M. Kamada N. (2020). The bacterial connection between the oral cavity and the gut diseases. J. Dent. Res. 99, 1021–1029. doi: 10.1177/0022034520924633 32464078 PMC7375741

[B29] Kotloff K. L. Winickoff J. P. Ivanoff B. Clemens J. D. Swerdlow D. L. Sansonetti P. J. (1999). Global burden of Shigella infections: implications for vaccine development and implementation of control strategies. Bull. World Health Organ. 77, 651–666. 10516787 PMC2557719

[B30] Kowalska-Duplaga K. Kapusta P. Gosiewski T. Sroka-Oleksiak A. Ludwig-Słomczyńska A. H. Wołkow P. P.. (2020). Changes in the intestinal microbiota are seen following treatment with infliximab in children with Crohn’s disease. J. Clin. Med. 9, 1–16. doi: 10.3390/jcm9030687 PMC7141282 32143438

[B31] Kucharzik T. Ellul P. Greuter T. Rahier J. F. Verstockt B. Abreu C.. (2021). ECCO guidelines on the prevention, diagnosis, and management of infections in inflammatory bowel disease. J. Crohns Colitis 15, 879–913. doi: 10.1093/ecco-jcc/jjab052 33730753

[B32] Lamb C. A. Kennedy N. A. Raine T. Hendy P. A. Smith P. J. Limdi J. K.. (2019). British Society of Gastroenterology consensus guidelines on the management of inflammatory bowel disease in adults. Gut 68, s1–s106. doi: 10.1136/gutjnl-2019-318484 31562236 PMC6872448

[B33] Lu J. Breitwieser F. P. Thielen P. Salzberg S. L. (2017). Bracken: Estimating species abundance in metagenomics data. PeerJ Comput. Sci. 2017, 1–17. doi: 10.7717/peerj-cs.104 PMC12016282 40271438

[B34] Luan Y. Y. Yao Y. M. (2018). The clinical significance and potential role of C-reactive protein in chronic inflammatory and neurodegenerative diseases. Front. Immunol. 9. doi: 10.3389/fimmu.2018.01302 PMC6008573 29951057

[B35] Ma L. Hou C. Yang H. Chen Q. Lyu W. Wang Z.. (2023). Multi-omics analysis reveals the interaction of gut microbiome and host microRNAs in ulcerative colitis. Ann. Med. 55, 1–13. doi: 10.1080/07853890.2023.2261477 37774039 PMC10543339

[B36] Mah C. Jayawardana T. Leong G. Koentgen S. Lemberg D. Connor S. J.. (2023). Assessing the relationship between the gut microbiota and inflammatory bowel disease therapeutics: A systematic review. Pathogens 12, 1–42. doi: 10.3390/pathogens12020262 PMC9965214 36839534

[B37] Majchrzak K. Fichna J. (2019). Biologic therapy in crohn’s disease–what we have learnt so far. Curr. Drug Targets 21, 792–806. doi: 10.2174/1389450121666191218123203 31854272

[B38] Marti-Aguado D. Ballester M. P. Bosca-Watts M. M. (2017). Invasive pulmonary aspergillosis in an immunocompromised patient with severe ulcerative colitis. Rev. Espanola Enfermedades Digestivas 109, 316–317. doi: 10.17235/reed.2017.4564/2016 28229616

[B39] Martin M. (2011). Cutadapt removes adapter sequences from high-throughput sequencing reads. EMBnet J. 17, 10–12. doi: 10.14806/ej.17.1.200

[B40] Miquel S. Martín R. Rossi O. Bermúdez-Humarán L. G. Chatel J. M. Sokol H.. (2013). Faecalibacterium prausnitzii and human intestinal health. Curr. Opin. Microbiol. 16, 255–261. doi: 10.1016/j.mib.2013.06.003 23831042

[B41] Mohebali N. Weigel M. Hain T. Sütel M. Bull J. Kreikemeyer B.. (2023). Faecalibacterium prausnitzii, Bacteroides faecis and Roseburia intestinalis attenuate clinical symptoms of experimental colitis by regulating Treg/Th17 cell balance and intestinal barrier integrity. Biomed. Pharmacother. 167, 1–15. doi: 10.1016/j.biopha.2023.115568 37793274

[B42] Narula N. Dhillon A. Zhang D. Sherlock M. E. Tondeur M. Zachos M. (2018). Enteral nutritional therapy for induction of remission in Crohn’s disease. Cochrane Database Systematic Rev. 2018, 1–68. doi: 10.1002/14651858.CD000542.pub3 PMC6494406 29607496

[B43] Nie K. Ma K. Luo W. Shen Z. Yang Z. Xiao M.. (2021). Roseburia intestinalis: A beneficial gut organism from the discoveries in genus and species. Front. Cell Infect. Microbiol. 11. doi: 10.3389/fcimb.2021.757718 PMC8647967 34881193

[B44] Nishino K. Nishida A. Inoue R. Kawada Y. Ohno M. Sakai S.. (2018). Analysis of endoscopic brush samples identified mucosa-associated dysbiosis in inflammatory bowel disease. J. Gastroenterol. 53, 95–106. doi: 10.1007/s00535-017-1384-4 28852861

[B45] Nomura R. Tsuzuki S. Kojima T. Nagasawa M. Sato Y. Uefune M.. (2022). Administration of Aspergillus oryzae suppresses DSS-induced colitis. Food Chem.: Mol. Sci. 4, 1–9. doi: 10.1016/j.fochms.2021.100063 PMC8991515 35415669

[B46] Norman J. M. Handley S. A. Baldridge M. T. Droit L. Liu C. Y. Keller B. C.. (2015). Disease-specific alterations in the enteric virome in inflammatory bowel disease. Cell 160, 447–460. doi: 10.1016/j.cell.2015.01.002 25619688 PMC4312520

[B47] Oksanen J. Simpson G. Blanchet F. Kindt R. Legendre P. Minchin P.. (2022). vegan: Community Ecology Package (Vienna, Austria: R package version 2.6-4).

[B48] Park S. K. Kim H. N. Choi C. H. Im J. P. Cha J. M. Eun C. S.. (2020). Differentially abundant bacterial taxa associated with prognostic variables of crohn′s disease: Results from the impact study. J. Clin. Med. 9, 1–17. doi: 10.3390/jcm9061748 PMC7357029 32516912

[B49] Parker B. J. Wearsch P. A. Veloo A. C. M. Rodriguez-Palacios A. (2020). The genus alistipes: gut bacteria with emerging implications to inflammation, cancer, and mental health. Front. Immunol. 11. doi: 10.3389/fimmu.2020.00906 PMC7296073 32582143

[B50] Parsaei M. Sarafraz N. Moaddab S. Y. Ebrahimzadeh Leylabadlo H. (2021). The importance of Faecalibacterium prausnitzii in human health and diseases. New Microbes New Infect. 43, 1–2. doi: 10.1016/j.nmni.2021.100928 PMC8365382 34430035

[B51] Pascal V. Pozuelo M. Borruel N. Casellas F. Campos D. Santiago A.. (2017). A microbial signature for Crohn’s disease. Gut 66, 813–822. doi: 10.1136/gutjnl-2016-313235 28179361 PMC5531220

[B52] Phalipon A. Sansonetti P. J. (2003). Shigellosis: innate mechanisms of inflammatory destruction of the intestinal epithelium, adaptive immune response, and vaccine development. Crit. Rev. Immunol. 23, 371–401. doi: 10.1615/CritRevImmunol.v23.i56.20 15030306

[B53] Qiu X. Ma J. Jiao C. Mao X. Zhao X. Lu M.. (2017). Alterations in the mucosa-associated fungal microbiota in patients with ulcerative colitis. Oncotarget 8, 107577–107588. doi: 10.18632/oncotarget.22534 29296188 PMC5746090

[B54] Raine T. Bonovas S. Burisch J. Kucharzik T. Adamina M. Annese V.. (2022). ECCO guidelines on therapeutics in ulcerative colitis: medical treatment. J. Crohns Colitis 16, 2–17. doi: 10.1093/ecco-jcc/jjab178 34635919

[B55] Rampelli S. Soverini M. Turroni S. Quercia S. Biagi E. Brigidi P.. (2016). ViromeScan: A new tool for metagenomic viral community profiling. BMC Genomics 17, 1–9. doi: 10.1186/s12864-016-2446-3 26932765 PMC4774116

[B56] Rautio M. Eerola E. Väisänen-Tunkelrott M. L. Molitoris D. Lawson P. Collins M. D.. (2003). Reclassification of Bacteroides putredinis (Weinberg et al., 1937) in a new genus Alistipes gen. nov., as Alistipes putredinis comb. nov., and description of Alistipes finegoldii sp. nov., from human sources. Syst. Appl. Microbiol. 26, 182–188. doi: 10.1078/072320203322346029 12866844

[B57] Rhodes J. M. (2007). The role of Escherichia coli in inflammatory bowel disease. Gut 56, 610–612. doi: 10.1136/gut.2006.111872 17440180 PMC1942130

[B58] Ribaldone D. G. Caviglia G. P. Abdulle A. Pellicano R. Ditto M. C. Morino M.. (2019). Adalimumab therapy improves intestinal dysbiosis in Crohn’s disease. J. Clin. Med. 8, 1–10. doi: 10.3390/jcm8101646 PMC6832711 31601034

[B59] Ricanek P. Lothe S. M. Frye S. A. Rydning A. Vatn M. H. Tønjum T. (2012). Gut bacterial profile in patients newly diagnosed with treatment-naïve Crohn’s disease. Clin. Exp. Gastroenterol. 5, 173–186. doi: 10.2147/CEG.S33858 23049264 PMC3459595

[B60] Ritchie M. E. Phipson B. Wu D. Hu Y. Law C. W. Shi W.. (2015). Limma powers differential expression analyses for RNA-sequencing and microarray studies. Nucleic Acids Res. 43, 1–13. doi: 10.1093/nar/gkv007 25605792 PMC4402510

[B61] Ruan G. Chen M. Chen L. Xu F. Xiao Z. Yi A.. (2022). Roseburia intestinalis and Its Metabolite Butyrate Inhibit Colitis and Upregulate TLR5 through the SP3 Signaling Pathway. Nutrients 14, 1–16. doi: 10.3390/nu14153041 PMC9332583 35893896

[B62] Said H. S. Suda W. Nakagome S. Chinen H. Oshima K. Kim S.. (2014). Dysbiosis of salivary microbiota in inflammatory bowel disease and its association with oral immunological biomarkers. DNA Res. 21, 15–25. doi: 10.1093/dnares/dst037 24013298 PMC3925391

[B63] Sakamoto M. Ikeyama N. Yuki M. Murakami T. Mori H. Iino T.. (2021). Adlercreutzia hattorii sp. Nov., an equol non-producing bacterium isolated from human faeces. Int. J. Syst. Evol. Microbiol. 71, 1–9. doi: 10.1099/ijsem.0.005121 34870581

[B64] Sakurai T. Saruta M. (2023). Positioning and usefulness of biomarkers in inflammatory bowel disease. Digestion 104, 30–41. doi: 10.1159/000527846 36404714 PMC9843547

[B65] Saraav I. Cervantes-Barragan L. Olias P. Fu Y. Wang Q. Wang L. (2021). Chronic Toxoplasma gondii infection enhances susceptibility to colitis. Proc. Natl. Acad. Sci. U. S. A. 118, e2106730118. doi: 10.1073/pnas.2106730118/-/DCSupplemental 34462359 PMC8433586

[B66] Sartor R. B. Wu G. D. (2017). Roles for intestinal bacteria, viruses, and fungi in pathogenesis of inflammatory bowel diseases and therapeutic approaches. Gastroenterology 152, 327–339.e4. doi: 10.1053/j.gastro.2016.10.012 27769810 PMC5511756

[B67] Schirmer M. Garner A. Vlamakis H. Xavier R. J. (2019). Microbial genes and pathways in inflammatory bowel disease. Nat. Rev. Microbiol. 17, 497–511. doi: 10.1038/s41579-019-0213-6 31249397 PMC6759048

[B68] Seidler Y. Rimbach G. Lüersen K. Vinderola G. Ipharraguerre I. R. (2024). The postbiotic potential of Aspergillus oryzae – a narrative review. Front. Microbiol. 15. doi: 10.3389/fmicb.2024.1452725 PMC11538067 39507340

[B69] Serrano-Gómez G. Mayorga L. Oyarzun I. Roca J. Borruel N. Casellas F.. (2021). Dysbiosis and relapse-related microbiome in inflammatory bowel disease: A shotgun metagenomic approach. Comput. Struct. Biotechnol. J. 19, 6481–6489. doi: 10.1016/j.csbj.2021.11.037 34938418 PMC8665270

[B70] Shan Y. Lee M. Chang E. B. (2022). The gut microbiome and inflammatory bowel diseases. Annu. Rev. Med. 73, 455–468. doi: 10.1146/annurev-med-042320-021020 34555295 PMC10012812

[B71] Shannon C. E. (1948). A mathematical theory of communication. Bell System Tech. J. 27, 379–423. doi: 10.1002/j.1538-7305.1948.tb01338.x

[B72] Sokol H. Leducq V. Aschard H. Pham H. P. Jegou S. Landman C.. (2017). Fungal microbiota dysbiosis in IBD. Gut 66, 1039–1048. doi: 10.1136/gutjnl-2015-310746 26843508 PMC5532459

[B73] Spinelli A. Bonovas S. Burisch J. Kucharzik T. Adamina M. Annese V.. (2022). ECCO guidelines on therapeutics in ulcerative colitis: surgical treatment. J. Crohns Colitis 16, 179–189. doi: 10.1093/ecco-jcc/jjab177 34635910

[B74] Tavakoli P. Vollmer-Conna U. Hadzi-Pavlovic D. Grimm M. C. (2021). A review of inflammatory bowel disease: A model of microbial, immune and neuropsychological integration. Public Health Rev. 42, 1–21. doi: 10.3389/phrs.2021.1603990 PMC8386758 34692176

[B75] Ternes D. Karta J. Tsenkova M. Wilmes P. Haan S. Letellier E. (2020). Microbiome in colorectal cancer: how to get from meta-omics to mechanism? Trends Microbiol. 28, 401–423. doi: 10.1016/j.tim.2020.01.001 32298617

[B76] Ungaro F. Massimino L. Furfaro F. Rimoldi V. Peyrin-Biroulet L. D’Alessio S.. (2019). Metagenomic analysis of intestinal mucosa revealed a specific eukaryotic gut virome signature in early-diagnosed inflammatory bowel disease. Gut Microbes 10, 149–158. doi: 10.1080/19490976.2018.1511664 30252582 PMC6546319

[B77] Vieujean S. Jairath V. Peyrin-Biroulet L. Dubinsky M. Iacucci M. Magro F.. (2025). Understanding the therapeutic toolkit for inflammatory bowel disease. Nat. Rev. Gastroenterol. Hepatol. 22, 371–394. doi: 10.1038/s41575-024-01035-7 39891014

[B78] Wagatsuma K. Yokoyama Y. Nakase H. (2021). Role of biomarkers in the diagnosis and treatment of inflammatory bowel disease. Life 11, 1–21. doi: 10.3390/life11121375 PMC8707558 34947906

[B79] Weyand N. J. (2017). Neisseria models of infection and persistence in the upper respiratory tract. Pathog. Dis. 75, 1–13. doi: 10.1093/femspd/ftx031 28369241

[B80] Wood D. E. Lu J. Langmead B. (2019). Improved metagenomic analysis with Kraken 2. Genome Biol. 20, 1–13. doi: 10.1186/s13059-019-1891-0 31779668 PMC6883579

[B81] Yu S. Sun Y. Shao X. Zhou Y. Yu Y. Kuai X.. (2022). Leaky gut in IBD: intestinal barrier–gut microbiota interaction. J. Microbiol. Biotechnol. 32, 825–834. doi: 10.4014/jmb.2203.03022 35791076 PMC9628915

[B82] Zhou F. X. Chen L. Liu X. W. Ouyang C. H. Wu X. P. Wang X. H.. (2012). Lactobacillus crispatus M206119 exacerbates murine DSScolitis by interfering with inflammatory responses. World J. Gastroenterol. 18, 2344–2356. doi: 10.3748/wjg.v18.i19.2344 22654425 PMC3353368

