# Supplementary material for: Exploration of fecal microbiota in newly diagnosed patients with inflammatory bowel disease using shotgun metagenomics
Source: Front Cell Infect Microbiol. 2025 Jul 1;15:1595884. doi: 10.3389/fcimb.2025.1595884 (PMC12259665; doi:10.3389/fcimb.2025.1595884)
Supplement: Supplementary file 1 [file Supplementaryfile1.docx]

Supplementary Material

# Supplementary Figures


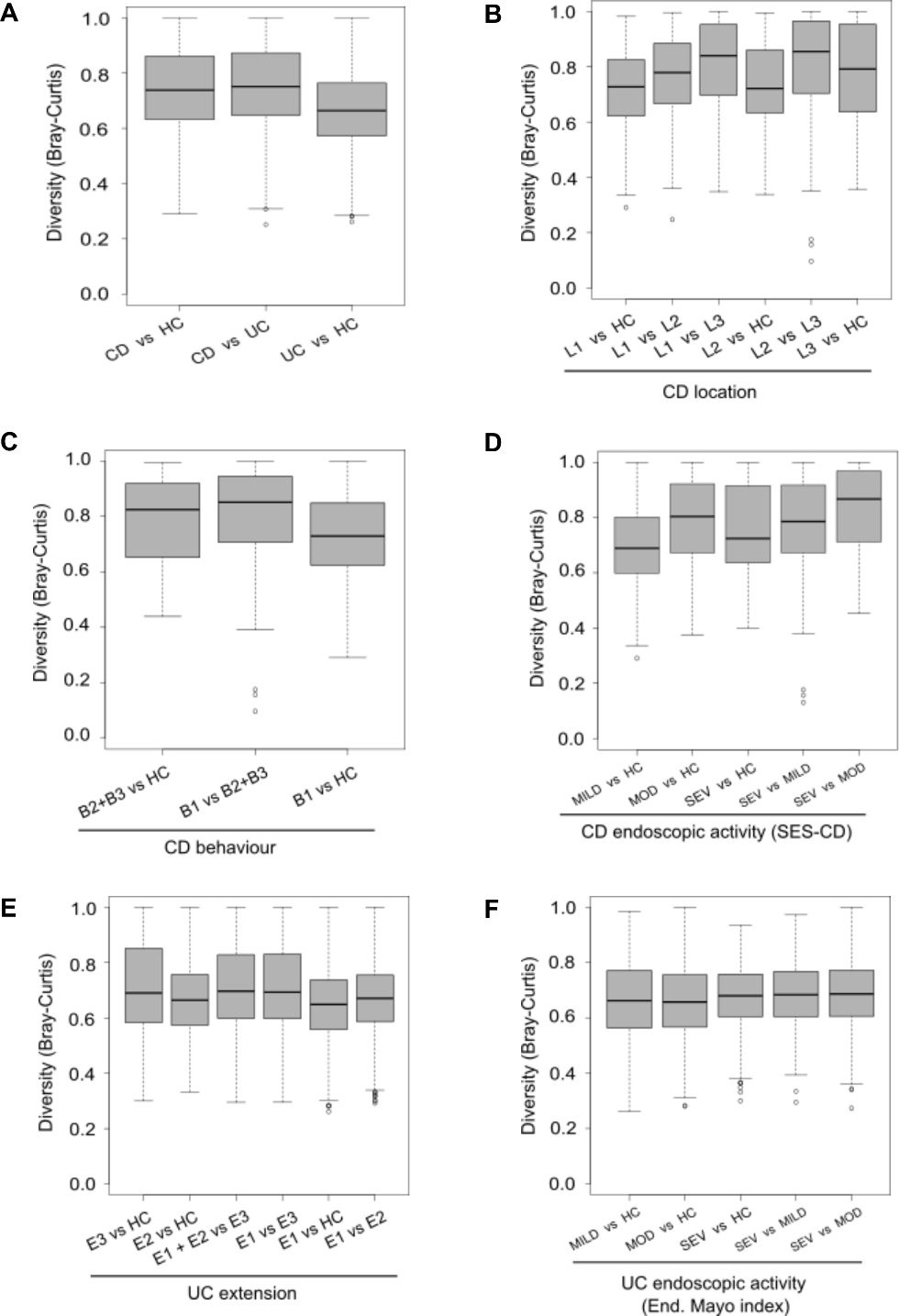


**Supplementary Figure 1. β-Diversity of intestinal flora in stool samples from the IBDomics cohort.** Panels A-F display β-diversity calculated using Bray-Curtis dissimilarity between pair-wise combinations based on: IBD type (A), CD location (B), CD behavior (C), CD severity (D), UC extension (E), and UC severity (F).


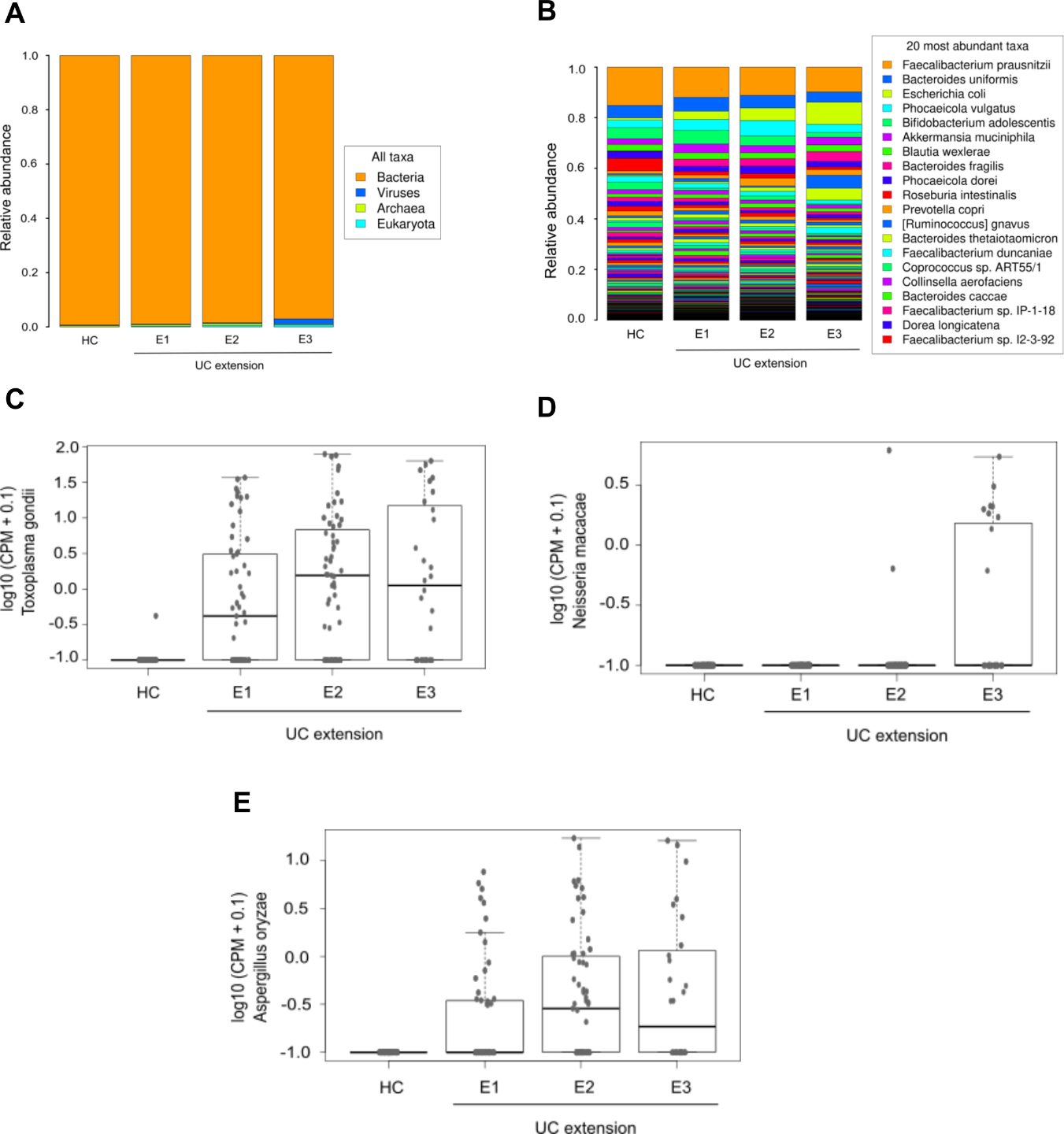


**Supplementary Figure 2. Taxonomic abundance in ulcerative colitis patients according to damage extension.** Stacked bars in both histograms illustrate the relative abundances of all domains (A) and the most common species (B) determined in the three UC patient groups defined according to damage location, and in HC. Panels C-E display a selection of species with significant differences (Kruskal-Wallis p_value < 0.05) between groups: *Toxoplasma gondii* (C), *Neisseria macacae* (D), and *Aspergillus oryzae* (E) levels, represented in counts per million.


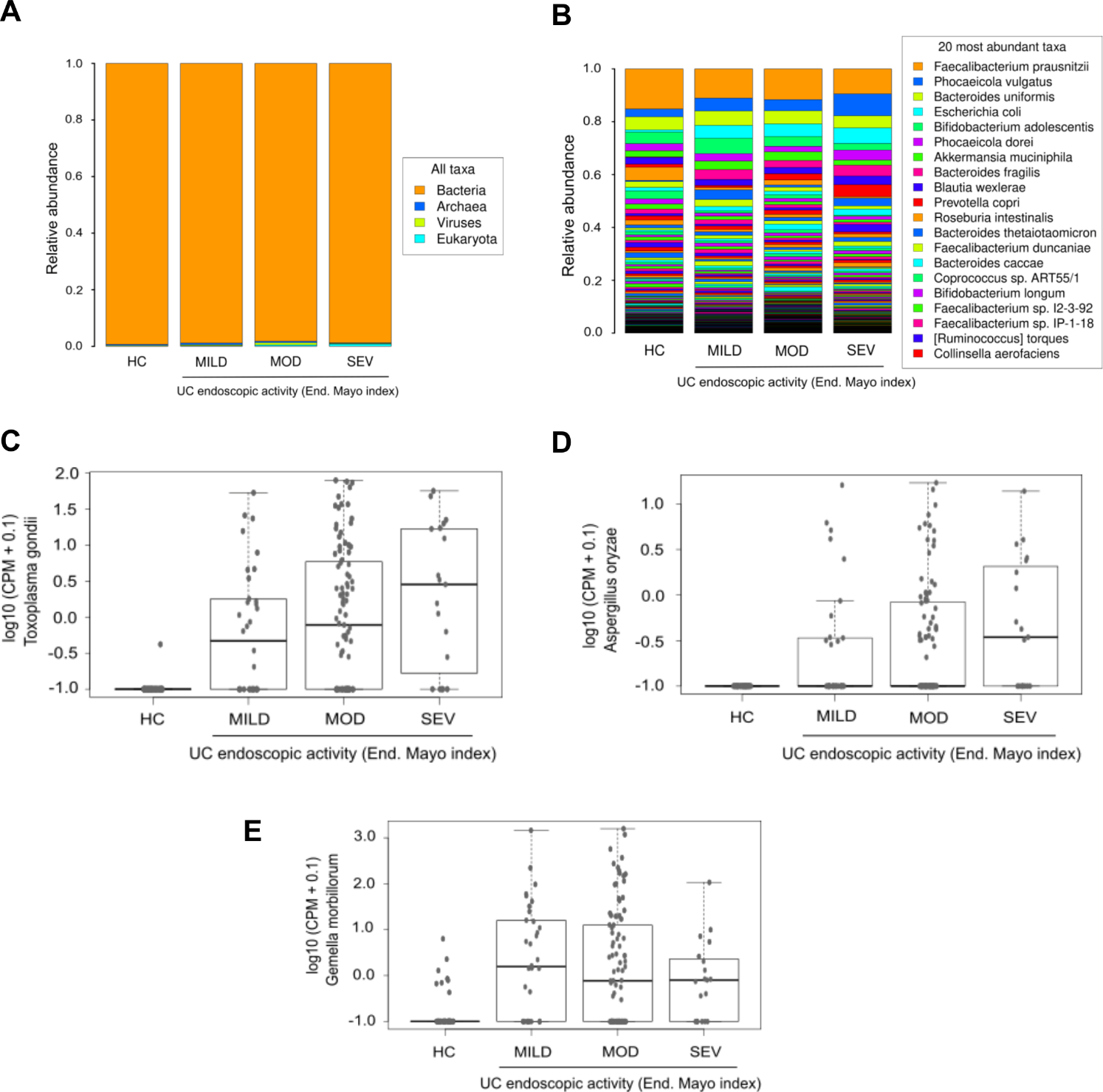


**Supplementary Figure 3. Abundance of microbiological species according to ulcerative colitis severity.** Histograms depict the relative abundances of all domains (A) and the most common species (B) identified in the three UC patient groups defined according to disease severity, and in HC. Graphs C-E illustrate a selection of species with significant differences (Kruskal-Wallis p_value < 0.05) between groups: *Toxoplasma gondii* (C), *Aspergillus oryzae* (D), and *Gemella morbillorum* (E) levels, represented in counts per million.

# Supplementary Tables.

**Supplementary Table 1. Reference genomes and species used in the custom mapping database.** The database used for taxonomic classification was generated with the Kraken2-build application and includes components for Refeq archaea, bacteria, viral, plasmid, human, UniVec_Core, protozoa and fungi. The TaxRank column uses one letter initials to represent levels ROOT, DOMAIN, KINGDOM, PHYLUM, CLASS, ORDER, FAMILY, GENUS, SPECIES, and numerical indices to represent sub-levels (Excel file "Supplementary_Table_1.xls").

**Supplementary Table 2.** Total pathways detected and pathways with the most significant differences in IBD patients compared to HC individuals, and between Crohn’s disease and ulcerative colitis patients. Abundance differences are expressed as fold changes (n-fold) between the first and the second conditions being compared, and are significant with adjusted p_value < 0.05, according to tests performed with limma.

| **Comparisons** | **Total pathways selected** | **Total pathways with significant differences** | **Top six pathways**  **with significant differences (n-fold)** |
| --- | --- | --- | --- |
| IBD vs HC | 399 | 256 | PWY-6901: superpathway of glucose and xylose degradation (5.0)  PWY0-1298: superpathway of pyrimidine deoxyribonucleosides degradation (4.6)  GLUCONEO-PWY: gluconeogenesis I (4.5)  PWY-7117: C4 photosynthetic carbon assimilation cycle PEPCK type (4.1)  PWY0-301: L-ascorbate degradation I bacterial anaerobic (4.0)  FAO-PWY: fatty acid beta-oxidation I generic (4.0) |
| CD vs HC | 399 | 272 | PWY0-1298: superpathway of pyrimidine deoxyribonucleosides degradation (5.7)  THISYNARA-PWY: superpathway of thiamine diphosphate biosynthesis III eukaryotes (-5.3)  PWY-6901: superpathway of glucose and xylose degradation (5.3)  PWY-841: superpathway of purine nucleotides de novo biosynthesis I (4.7)  P161-PWY: acetylene degradation anaerobic (4.7)  GLUCONEO-PWY: gluconeogenesis I (4.6) |
| UC vs HC | 399 | 215 | PWY-6901: superpathway of glucose and xylose degradation (4.8)  GLUCONEO-PWY: gluconeogenesis I (4.2)  PWY-5723: Rubisco shunt (3.9)  PWY-7117: C4 photosynthetic carbon assimilation cycle PEPCK type (3.9)  GLYCOLYSIS-E-D: superpathway of glycolysis and the Entner-Doudoroff pathway (3.9)  FAO-PWY: fatty acid beta-oxidation I generic (3.8) |
| CD vs UC | 399 | 74 | THISYNARA-PWY: superpathway of thiamine diphosphate biosynthesis III eukaryotes (-3.8)  PWY-2942: L-lysine biosynthesis III (-3.1)  PWY-5030: L-histidine degradation III (-2.8)  HISDEG-PWY: L-histidine degradation I (-2.4)  P164-PWY: purine nucleobases degradation I anaerobic (2.4)  METHANOGENESIS-PWY: methanogenesis from H2 and CO2 (-2.2) |

HC: healthy controls; CD: Crohn´s disease; UC: ulcerative colitis.

**Supplementary Table 3.** Total pathways detected and pathways with the most significant differences in Crohn´s disease patients according to disease location. Abundance differences are expressed as fold changes (n-fold) between the first and the second conditions being compared, and are significant with adjusted p_value < 0.05, according to tests performed with limma.

| **Comparisons** | **Total pathways selected** | **Total pathways with significant differences** | **Top six pathways**  **with significant differences (n-fold)** |
| --- | --- | --- | --- |
| L1 vs HC | 390 | 170 | PWY0-1298: superpathway of pyrimidine deoxyribonucleosides degradation (4.4)  PWY-6901: superpathway of glucose and xylose degradation (4.3)  P161-PWY: acetylene degradation anaerobic (3.8)  GLUCONEO-PWY: gluconeogenesis I (3.2)  PWY-841: superpathway of purine nucleotides de novo biosynthesis I (3.2)  PWY-7356: thiamine diphosphate salvage IV yeast (-3.1) |
| L2 vs HC | 390 | 262 | THISYNARA-PWY: superpathway of thiamine diphosphate biosynthesis III eukaryotes (-8.0)  PWY-5676: acetyl-CoA fermentation to butanoate II (-7.7)  METH-ACETATE-PWY: methanogenesis from acetate (-7.2)  PWY-1861: formaldehyde assimilation II assimilatory RuMP Cycle (-7.2)  PWY-2942: L-lysine biosynthesis III (-6.8)  PWY-6901: superpathway of glucose and xylose degradation (6.8) |
| L3 vs HC | 390 | 252 | THISYNARA-PWY: superpathway of thiamine diphosphate biosynthesis III eukaryotes (-6.8)  PWY0-1298: superpathway of pyrimidine deoxyribonucleosides degradation (6.0)  PWY0-301: L-ascorbate degradation I bacterial anaerobic (5.7)  POLYISOPRENSYN-PWY: polyisoprenoid biosynthesis E. coli (5.7)  PWY-2942: L-lysine biosynthesis III (-5.7)  HISDEG-PWY: L-histidine degradation I (-5.3) |
| L1 vs L2 | 390 | 121 | PWY-1861: formaldehyde assimilation II assimilatory RuMP Cycle (6.1)  PWY-5676: acetyl-CoA fermentation to butanoate II (5.6)  METH-ACETATE-PWY: methanogenesis from acetate (5.6)  PWY-2942: L-lysine biosynthesis III (5.6)  THISYNARA-PWY: superpathway of thiamine diphosphate biosynthesis III eukaryotes (5.3)  PWY-8131: 5'-deoxyadenosine degradation II (5.0) |
| L1 vs L3 | 390 | 45 | HISDEG-PWY: L-histidine degradation I (4.5)  PWY-2942: L-lysine biosynthesis III (4.5)  THISYNARA-PWY: superpathway of thiamine diphosphate biosynthesis III eukaryotes (4.1)  PWY-6284: superpathway of unsaturated fatty acids biosynthesis E. coli (-3.9)  PWY-5367: petroselinate biosynthesis (-3.8)  PWY-7013: S-propane-12-diol degradation (3.4) |
| L2 vs L3 | 390 | 11 | HCAMHPDEG-PWY: 3-phenylpropanoate and 3-3-hydroxyphenylpropanoate degradation to 2-hydroxypentadienoate (4.9)  PWY-6690: cinnamate and 3-hydroxycinnamate degradation to 2-hydroxypentadienoate (4.9)  PWY-1861: formaldehyde assimilation II assimilatory RuMP Cycle (-4.8)  PWY4LZ-257: superpathway of fermentation Chlamydomonas reinhardtii (4.8)  PWY0-1277: 3-phenylpropanoate and 3-3-hydroxyphenylpropanoate degradation (4.7)  ARGDEG-PWY: superpathway of L-arginine putrescine and 4-aminobutanoate_degradation (4.5) |

HC: healthy controls; L1: ileal; L2: colonic; L3: ileocolonic.

**Supplementary Table 4.** Total pathways detected and pathways with the most significant differences in Crohn’s disease patients according to disease behavior. Abundance differences are expressed as fold changes (n-fold) between the first and the second conditions being compared, and are significant with adjusted p_value < 0.05, according to tests performed with limma.

| **Comparisons** | **Total pathways selected** | **Total pathways with significant differences** | **Top six pathways**  **with significant differences (n-fold)** |
| --- | --- | --- | --- |
| B1 vs HC | 390 | 266 | PWY0-1298: superpathway of pyrimidine deoxyribonucleosides degradation (5.0)  PWY-6901: superpathway of glucose and xylose degradation (4.8)  THISYNARA-PWY: superpathway of thiamine diphosphate biosynthesis III eukaryotes (-4.3)  PWY0-301: L-ascorbate degradation I bacterial anaerobic (4.2)  POLYISOPRENSYN-PWY: polyisoprenoid biosynthesis E. coli (4.2)  GLUCONEO-PWY: gluconeogenesis I (4.2) |
| B2 + B3 vs HC | 390 | 175 | THISYNARA-PWY: superpathway of thiamine diphosphate biosynthesis III eukaryotes (-12.1)  PWY-5676: acetyl-CoA fermentation to butanoate II (-8.6)  PWY-2942: L-lysine biosynthesis III (-8.3)  PWY-1861: formaldehyde assimilation II assimilatory RuMP Cycle (-8.2)  METH-ACETATE-PWY: methanogenesis from acetate (-8.0)  PWY-4984: urea cycle (-7.0) |
| B1 vs B2 + B3 | 390 | 11 | THISYNARA-PWY: superpathway of thiamine diphosphate biosynthesis III eukaryotes (7.8)  PWY-1861: formaldehyde assimilation II assimilatory RuMP Cycle (6.3)  PWY-8131: 5'-deoxyadenosine degradation II (6.1)  PWY-7013: S-propane-12-diol degradation (6.0)  METH-ACETATE-PWY: methanogenesis from acetate (5.4)  PWY-2942: L-lysine biosynthesis III (5.3) |

HC: healthy controls; B1: inflammatory; B2: stricturing; B3: penetrating

**Supplementary Table 5.** Total pathways detected and pathways with the most significant differences in Crohn´s disease patients according to endoscopic severity. Abundance differences are expressed as fold changes (n-fold) between the first and the second conditions being compared, and are significant with adjusted p_value < 0.05, according to tests performed with limma.

| **Comparisons** | **Total pathways selected** | **Total pathways with significant differences** | **Top six pathways**  **with significant differences (n-fold)** |
| --- | --- | --- | --- |
| Mild vs HC | 383 | 252 | PWY-6901: superpathway of glucose and xylose degradation (5.4)  METH-ACETATE-PWY: methanogenesis from acetate (-5.3)  PWY-5676: acetyl-CoA fermentation to butanoate II (-5.1)  PWY0-1298: superpathway of pyrimidine deoxyribonucleosides degradation (4.8)  PWY-4984: urea cycle (-4.7)  THISYNARA-PWY: superpathway of thiamine diphosphate biosynthesis III eukaryotes (-4,7) |
| Moderate vs HC | 383 | 156 | PWY0-1298: superpathway of pyrimidine deoxyribonucleosides degradation (5.7)  P164-PWY: purine nucleobases degradation I anaerobic (5.0)  PWY66-409: superpathway of purine nucleotide salvage (5.0)  POLYISOPRENSYN-PWY: polyisoprenoid biosynthesis E. coli (4.8)  METHANOGENESIS-PWY: methanogenesis from H2 and CO2 (-4.6)  PWY-5198: factor 420 biosynthesis II mycobacteria (-4.5) |
| Severe vs HC | 383 | 154 | PWY-6901: superpathway of glucose and xylose degradation (6.3)  PWY0-1298: superpathway of pyrimidine deoxyribonucleosides degradation (5.5)  POLYISOPRENSYN-PWY: polyisoprenoid biosynthesis E. coli (5.1)  PWY-7456: beta-14-mannan degradation (-5.1)  PWY-7117: C4 photosynthetic carbon assimilation cycle PEPCK type (5.1)  ORNDEG-PWY: superpathway of ornithine degradation (5.0) |
| Severe vs Mild | 383 | 0 |  |
| Severe vs Moderate | 383 | 0 |  |

HC: healthy controls.

**Supplementary Table 6.** Total pathways detected and pathways with the most significant differences in ulcerative colitis patients according to disease extension. Abundance differences are expressed as fold changes (n-fold) between the first and the second conditions being compared, and are significant with adjusted p_value < 0.05, according to tests performed with limma.

| **Comparisons** | **Total pathways selected** | **Total pathways with significant differences** | **Top six pathways**  **with significant differences (n-fold)** |
| --- | --- | --- | --- |
| E1 vs HC | 379 | 164 | PWY-6901: superpathway of glucose and xylose degradation (3.7)  GLUCONEO-PWY: gluconeogenesis I (3.4)  FAO-PWY: fatty acid beta-oxidation I generic (2.9)  PWY-5484: glycolysis II from fructose 6-phosphate (2.8)  BIOTIN-BIOSYNTHESIS-PWY: biotin biosynthesis I (2.8)  GLUCOSE1PMETAB-PWY: glucose and glucose-1-phosphate degradation (2.8) |
| E2 vs HC | 379 | 248 | PWY-6901: superpathway of glucose and xylose degradation (5.6)  GLYCOLYSIS-E-D: superpathway of glycolysis and the Entner-Doudoroff pathway (4.8)  PWY-5723: Rubisco shunt (4.8)  P441-PWY: superpathway of N-acetylneuraminate degradation (4.6)  PWY-5913: partial TCA cycle obligate autotrophs (4.6)  PWY-7117: C4 photosynthetic carbon assimilation cycle PEPCK type (4.5) |
| E3 vs HC | 379 | 283 | PWY-6901: superpathway of glucose and xylose degradation (6.2)  PWY-5723: Rubisco shunt (5.7)  ARG_POLYAMINE-SYN: superpathway of arginine and polyamine biosynthesis (5.7)  GLYCOLYSIS-E-D: superpathway of glycolysis and the Entner-Doudoroff pathway (5.6)  P441-PWY: superpathway of N-acetylneuraminate degradation (5.5)  BIOTIN-BIOSYNTHESIS-PWY: biotin biosynthesis I (5.5) |
| E1 vs E2 | 379 | 0 |  |
| E1 vs E3 | 379 | 213 | PWY-7345: superpathway of anaerobic sucrose degradation (-3.7)  PWY-7184: pyrimidine deoxyribonucleotides de novo biosynthesis I (-3.5)  PWY0-166: superpathway of pyrimidine deoxyribonucleotides de novo biosynthesis E. coli (-3.5)  DENOVOPURINE2-PWY: superpathway of purine nucleotides de novo biosynthesis II (-3.4)  PWY-5675: nitrate reduction V assimilatory (-3.3)  PWY-5837: 2-carboxy-14-naphthoquinol biosynthesis (-3.2) |
| E1 + E2 vs E3 | 376 | 85 | PWY-5837: 2-carboxy-14-naphthoquinol biosynthesis (-2.8)  HEME-BIOSYNTHESIS-II: heme b biosynthesis I aerobic (-2.7)  PWY66-409: superpathway of purine nucleotide salvage (-2.7)  DENOVOPURINE2-PWY: superpathway of purine nucleotides de novo biosynthesis II (-2.7)  PWY0-166: superpathway of pyrimidine deoxyribonucleotides de novo biosynthesis E. coli (-2.6)  PWY-7184: pyrimidine deoxyribonucleotides de novo biosynthesis I (-2.6) |

HC: healthy controls; E1: proctitis; E2: Left sided; E3: Extensive.

**Supplementary Table 7.** Total pathways detected and pathways with the most significant differences in ulcerative colitis patients according to endoscopic severity. Abundance differences are expressed as fold changes (n-fold) between the first and the second conditions being compared, and are significant with adjusted p_value < 0.05, according to tests performed with limma.

| **Comparisons** | **Total pathways selected** | **Total pathways with significant differences** | **Top six pathways**  **with significant differences (n-fold)** |
| --- | --- | --- | --- |
| Mild vs HC | 379 | 220 | PWY-6901: superpathway of glucose and xylose degradation (5.6)  GLUCONEO-PWY: gluconeogenesis I (4.3)  PWY-5723: Rubisco shunt (4.3)  KETOGLUCONMET-PWY: ketogluconate metabolism (4.2)  FAO-PWY: fatty acid beta-oxidation I generic (4.1)  HEXITOLDEGSUPER-PWY: superpathway of hexitol degradation bacteria (4.0) |
| Moderate vs HC | 379 | 247 | PWY-6901: superpathway of glucose and xylose degradation (4.9)  GLYCOLYSIS-E-D: superpathway of glycolysis and the Entner-Doudoroff pathway (4.3)  PWY-5723: Rubisco shunt (4.2)  BIOTIN-BIOSYNTHESIS-PWY: biotin biosynthesis I (4.2)  ORNDEG-PWY: superpathway of ornithine degradation (4.1)  PWY-5913: partial TCA cycle obligate autotrophs (4.1) |
| Severe vs HC | 379 | 195 | PWY-6901: superpathway of glucose and xylose degradation (5.1)  GLUCONEO-PWY: gluconeogenesis I (5.0)  GLUCOSE1PMETAB-PWY: glucose and glucose-1-phosphate degradation (4.4)  POLYAMSYN-PWY: superpathway of polyamine biosynthesis I (4.4)  BIOTIN-BIOSYNTHESIS-PWY: biotin biosynthesis I (4.4)  PWY-5723: Rubisco shunt (4.3) |
| Severe vs Mild | 379 | 0 |  |
| Severe vs Moderate | 379 | 0 |  |

HC: healthy controls.
